# Identification of Novel Mobilized Colistin Resistance Gene *mcr-9* in a Multidrug-Resistant, Colistin-Susceptible Salmonella enterica Serotype Typhimurium Isolate

**DOI:** 10.1128/mBio.00853-19

**Published:** 2019-05-07

**Authors:** Laura M. Carroll, Ahmed Gaballa, Claudia Guldimann, Genevieve Sullivan, Lory O. Henderson, Martin Wiedmann

**Affiliations:** a Department of Food Science, Cornell University, Ithaca, New York, USA; b Institute for Food Safety and Hygiene, University of Zurich, Zurich, Switzerland; c Department of Microbiology, Cornell University, Ithaca, New York, USA; University of Queensland; Eurofins; University of Tennessee

**Keywords:** Salmonella enterica, antibiotic resistance, colistin, *mcr* genes, *mcr-9*, mobilized colistin resistance, multidrug resistance, plasmid-mediated resistance

## Abstract

Mobilized colistin resistance (*mcr*) genes are plasmid-borne genes that confer resistance to colistin, an antibiotic used to treat severe bacterial infections. To date, eight known *mcr* homologues have been described (*mcr-1* to *-8*). Here, we describe *mcr-9*, a novel *mcr* homologue detected during routine *in silico* screening of sequenced *Salmonella* genomes for antimicrobial resistance genes. The amino acid sequence of *mcr-9*, detected in a multidrug-resistant (MDR) Salmonella enterica serotype Typhimurium (*S*. Typhimurium) strain isolated from a human patient in Washington State in 2010, most closely resembled *mcr-3*, aligning with 64.5% amino acid identity and 99.5% coverage using Translated Nucleotide BLAST (tblastn). The *S.* Typhimurium strain was tested for phenotypic resistance to colistin and was found to be sensitive at the 2-mg/liter European Committee on Antimicrobial Susceptibility Testing breakpoint under the tested conditions. *mcr-9* was cloned in colistin-susceptible Escherichia coli NEB5α under an IPTG (isopropyl-β-d-thiogalactopyranoside)-induced promoter to determine whether it was capable of conferring resistance to colistin when expressed in a heterologous host. Expression of *mcr-9* conferred resistance to colistin in E. coli NEB5α at 1, 2, and 2.5 mg/liter colistin, albeit at a lower level than *mcr-3*. Pairwise comparisons of the predicted protein structures associated with all nine *mcr* homologues (Mcr-1 to -9) revealed that Mcr-9, Mcr-3, Mcr-4, and Mcr-7 share a high degree of similarity at the structural level. Our results indicate that *mcr-9* is capable of conferring phenotypic resistance to colistin in Enterobacteriaceae and should be immediately considered when monitoring plasmid-mediated colistin resistance.

## Abstract (precis)

Colistin is a last-resort antibiotic that is used to treat severe infections caused by MDR and extensively drug-resistant (XDR) bacteria. The World Health Organization (WHO) has designated colistin as a “highest priority critically important antimicrobial for human medicine” (WHO, *Critically Important Antimicrobials for Human Medicine*, *5th revision*, 2017, https://www.who.int/foodsafety/publications/antimicrobials-fifth/en/), as it is often one of the only therapies available for treating serious bacterial infections in critically ill patients. Plasmid-borne *mcr* genes that confer resistance to colistin pose a threat to public health at an international scale, as they can be transmitted via horizontal gene transfer and have the potential to spread globally. Therefore, the establishment of a complete reference of *mcr* genes that can be used to screen for plasmid-mediated colistin resistance is essential for developing effective control strategies.

## OBSERVATION

Until recently, bacterial resistance to colistin, a last-resort antibiotic reserved for treating severe infections, was thought to be acquired solely via chromosomal point mutations (1). However, in 2015, plasmid-mediated colistin resistance gene *mcr-1* was described in Escherichia coli (1). Mcr-1 is a phosphoethanolamine transferase that modifies cell membrane lipid A head groups with a phosphoethanolamine residue, reducing affinity to colistin (2). Since then, seven additional *mcr* homologues (*mcr-2* to *-8*) have been identified in Enterobacteriaceae (3–9). Here, we report novel *mcr* homologue *mcr-9*, which was identified in a Salmonella enterica serotype Typhimurium (*S.* Typhimurium) genome.

### *In silico* identification of *mcr-9* in an MDR *S.* Typhimurium genome.

MDR *S*. Typhimurium strain HUM_TYPH_WA_10_R9_3274 (NCBI RefSeq accession no. GCF_002091095.1) was isolated from a patient in Washington State in 2010 (10). It had previously been tested for resistance to a panel of 12 antimicrobials that did not include colistin (10). ABRicate version 0.8 (https://github.com/tseemann/abricate) identified 20 antimicrobial resistance (AMR) genes in the HUM_TYPH_WA_10_R9_3274 assembly using the ResFinder database (accessed 11 June 2018) (11) and minimum identity and coverage thresholds of 75 and 50% (10), respectively, none of which had been previously described to confer colistin resistance (see Table S1 in the supplemental material). Four plasmid replicons, including IncHI2 and IncHI2A, were detected with at least 80% identity and 60% coverage using ABRicate and PlasmidFinder (accessed 11 June 2018 [Table S1]) (12).

10.1128/mBio.00853-19.4 TABLE S1 Antimicrobial resistance (AMR) genes and plasmid replicons detected in the assembly of HUM_TYPH_WA_10_R9_3274. Download Table S1, DOCX file, 0.1 MB. Copyright © 2019 Carroll et al. 2019 Carroll et al. This content is distributed under the terms of the Creative Commons Attribution 4.0 International license.

To detect *mcr-9* in the HUM_TYPH_WA_10_R9_3274 assembly, all colistin resistance-conferring nucleotide sequences available in ResFinder (52 sequences, accessed 22 January 2019 [see Table S2 in the supplemental material]) were translated into amino acid sequences using EMBOSS Transeq (reading frame 1 [https://www.ebi.ac.uk/Tools/st/emboss_transeq/]). The implementation of Translated Nucleotide BLAST (tblastn) (13) in BTyper version 2.3.2 (14) selected *mcr-3.17* as the highest-scoring *mcr* allele, which aligned to *mcr-9* with 64.5% amino acid identity and 99.5% coverage (Table S1).

10.1128/mBio.00853-19.5 TABLE S2 Comparison of *mcr-9* to previously published *mcr* homologues using Protein BLAST (blastp). Download Table S2, DOCX file, 0.1 MB. Copyright © 2019 Carroll et al. 2019 Carroll et al. This content is distributed under the terms of the Creative Commons Attribution 4.0 International license.

MUSCLE version 3.8.31 (15) was used to construct alignments of the amino acid sequence of *mcr-9* (NCBI protein accession no. WP_001572373.1) and the following: (i) the 52 *mcr* amino acid sequences from ResFinder (53 sequences [Table S2]), (ii) the top 100 hits produced when *mcr-9* was queried against NCBI’s non-redundant protein (nr) database using the Protein BLAST (blastp) web server (https://blast.ncbi.nlm.nih.gov/Blast.cgi?PAGE=Proteins [accessed 22 January 2019]; 152 sequences excluding *mcr-9*’s self-match [see Table S3 in the supplemental material]), and (iii) amino acid sequences of 61 putative phosphoethanolamine transferases used in other papers describing novel *mcr* genes (4, 5, 8, 9) (213 sequences [see Table S4 in the supplemental material]). For each alignment, RAxML version 8.2.12 (16) was used to construct a phylogeny using the PROTGAMMAAUTO method and 1,000 bootstrap replicates.

10.1128/mBio.00853-19.6 TABLE S3 Top 100 hits obtained by querying *mcr-9* against NCBI’s non-redundant protein sequence (nr) database using Protein BLAST (blastp). Download Table S3, DOCX file, 0.1 MB. Copyright © 2019 Carroll et al. 2019 Carroll et al. This content is distributed under the terms of the Creative Commons Attribution 4.0 International license.

10.1128/mBio.00853-19.7 TABLE S4 Comparison of *mcr-9* to putative phosphoethanolamine transferases used in other papers describing novel *mcr* genes. Download Table S4, DOCX file, 0.1 MB. Copyright © 2019 Carroll et al. 2019 Carroll et al. This content is distributed under the terms of the Creative Commons Attribution 4.0 International license.

**FIG 1:**
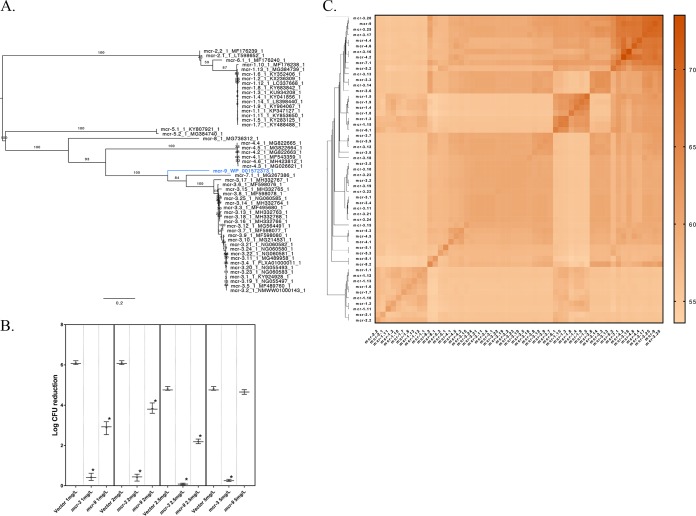
(A) Comparison of *mcr-9* to all previously described *mcr* homologues, based on amino acid sequence. The maximum likelihood phylogeny was constructed using RAxML version 8.2.12 with the amino acid sequences of novel mobilized colistin resistance gene *mcr-9* (in blue) and all previously described *mcr* genes (*mcr-1* to *-8* [in black]). The phylogeny is rooted at the midpoint, with branch lengths reported in substitutions per site. Branch labels correspond to bootstrap support percentages out of 1,000 replicates. (B) Colistin killing assay of E. coli NEB5α harboring a pLIV2 empty vector (negative control), *mcr-3* (positive control), or *mcr-9*, expressed under the control of the IPTG-controlled SPAC/lacOid promoter. Cells were grown in MH-II (Mueller-Hinton II) medium with IPTG to the mid-exponential phase. Colistin was added at concentrations of 0, 1, 2, 2.5, or 5 mg/liter, and the bacteria were incubated at 37°C for 1 h. The samples were diluted in phosphate-buffered saline (PBS) and plated on LB agar plates for the determination of CFU. Log CFU reduction was calculated by comparing CFU after each treatment to CFU levels obtained at 0 mg/liter colistin, using three independent biological replicates. Asterisks denote significant differences compared to empty vector treatment (*P* < 0.05 by Student's *t* test relative to the concentration's respective negative control after a Bonferroni correction). (C) Similarity matrix (composed of Dali *Z*-scores) of all previously described Mcr groups (Mcr-1 to -8) and Mcr-9, based on protein structure. The Dali server was used to perform all-against-all comparisons of 3D structural models based on all *mcr* homologues (Fig. 2A); for this analysis, amino acid sequences of *mcr-5.3* and *mcr-8.2*, which were not available in ResFinder, were additionally included from the National Database of Antibiotic Resistant Organisms (NDARO).

The amino acid sequence of *mcr-9* most closely resembled those of *mcr-3* and *mcr-7* (Fig. 1A; see Fig. S1 in the supplemental material). However, the *S.* Typhimurium isolate in which *mcr-9* was detected was not resistant to colistin at the >2-mg/liter European Committee on Antimicrobial Susceptibility Testing (EUCAST [http://www.eucast.org]) breakpoint when a broth microdilution method was used to determine the colistin MIC (see Table S5 in the supplemental material).

10.1128/mBio.00853-19.2 FIG S1 Maximum likelihood phylogenies constructed using the amino acid sequences of novel mobilized colistin resistance gene *mcr-9* (in blue) plus all (*n* = 52) previously described *mcr* genes (*mcr*-*1*, *-2*, *-3*, *-4*, *-5*, *-6*, *-7*, and *-8*) available in ResFinder (accessed 22 January 2019 [in pink]), as well as (A) the 100 top hits produced when *mcr-9* was queried against NCBI’s non-redundant protein sequence (nr) database using the Protein BLAST (blastp) web server (accessed 22 January 2019) and default parameters (152 total sequences; *mcr-9*’s self-match was excluded, as it was already present in the nr database), as well as (B) amino acid sequences of 61 putative phosphoethanolamine transferases used in other papers describing novel *mcr* genes (213 total sequences). RAxML version 8.2.12 was used to construct the phylogenies, which were annotated with FigTree version 1.4.3. The phylogenies are rooted at the midpoint, with branch lengths reported in substitutions per site. Branch labels correspond to bootstrap support percentages out of 1,000 replicates. Download FIG S1, PDF file, 0.1 MB. Copyright © 2019 Carroll et al. 2019 Carroll et al. This content is distributed under the terms of the Creative Commons Attribution 4.0 International license.

10.1128/mBio.00853-19.8 TABLE S5 MIC profiles of colistin against *Salmonella* strains tested in this study. Download Table S5, DOCX file, 0.1 MB. Copyright © 2019 Carroll et al. 2019 Carroll et al. This content is distributed under the terms of the Creative Commons Attribution 4.0 International license.

### *mcr-9* confers resistance to colistin when cloned into colistin-susceptible E. coli NEB5α.

Coding regions of *mcr-9* and *mcr-3* were cloned under the control of an IPTG (isopropyl-β-d-thiogalactopyranoside)-induced SPAC/lacOid promoter and expressed in E. coli NEB5α (see Text S1 in the supplemental material). Colistin killing assays (Fig. 1B; see Fig. S2 in the supplemental material) were performed by incubating E. coli harboring the empty pLIV2 vector (negative control), pLIV2 with *mcr-3* (positive control), or pLIV2 with *mcr-9* with different concentrations of colistin (0, 1, 2, 2.5, and 5 mg/liter). E. coli cells harboring the empty vector failed to survive at all tested colistin concentrations >0 mg/liter. While *mcr-3* expression conferred clinical levels of colistin resistance (i.e., beyond the 2-mg/liter EUCAST breakpoint) in E. coli at all tested concentrations, *mcr-9* expression conferred clinical resistance at 1, 2, and 2.5 mg/liter, but not 5 mg/liter of colistin (Fig. 1B; Fig. S2).

10.1128/mBio.00853-19.1 TEXT S1 Detailed descriptions of experimental methods. Download Text S1, DOCX file, 0.1 MB. Copyright © 2019 Carroll et al. 2019 Carroll et al. This content is distributed under the terms of the Creative Commons Attribution 4.0 International license.

10.1128/mBio.00853-19.3 FIG S2 Selected images associated with the colistin killing assay of E. coli NEB5α harboring a pLIV2 empty vector (negative control), *mcr-3* (positive control), or *mcr-9*, expressed under the control of the IPTG-controlled SPAC/lacOid promoter. Cells were grown in MH-II (Mueller-Hinton II) medium with IPTG to the mid-exponential phase. Colistin was added at concentrations of 0, 1, 2, 2.5, or 5 mg/liter, and the bacteria were incubated at 37°C for 1 h. The samples were diluted in PBS and plated on LB agar plates for the determination of CFU. Download FIG S2, PDF file, 2.4 MB. Copyright © 2019 Carroll et al. 2019 Carroll et al. This content is distributed under the terms of the Creative Commons Attribution 4.0 International license.

### Mcr-3, Mcr-4, Mcr-7, and Mcr-9 are highly similar at the structural level.

**FIG 2:**
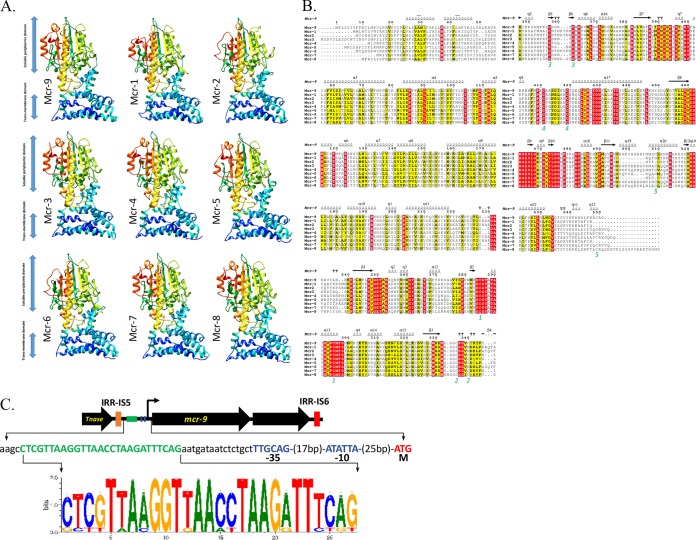
(A) Structural models of all published Mcr proteins (Mcr-1 to -8) and Mcr-9, based on lipooligosaccharide phosphoethanolamine transferase EptA. Models were constructed using the Phyre2 server, and structures were viewed and edited using UCSF Chimera. Structural models show conservation of two EptA domains: transmembrane-anchored and soluble periplasmic domains. (B) Location of Mcr-9 secondary structure elements within the alignment of Mcr amino acid sequences, constructed using the ESPript 3 server. The top track denotes Mcr-9 secondary structure elements (alpha helixes and beta sheets). Green digits below the alignment denote cysteine residues forming a disulfide bridge (e.g., 1 forms a bridge with 1, 2 with 2, etc.). Within the amino acid sequence alignment itself, a strict identity (i.e., identical amino acid residue at a site) is denoted by a red box and a white character. A yellow box around an amino acid residue denotes similarity across groups, where groups were defined using the default “all” specification in ESPript 3 (ESPript 3 total score [TSc] > in-group threshold [ThIn]), while a residue in boldface denotes similarity within a group (ESPript 3 in-group score [ISc] > ThIn). (C) Organization of the *mcr-9* locus in *S.* Typhimurium. An unknown function cupin fold metalloprotein is encoded by the gene downstream of *mcr-9* (unlabeled black arrow). The *mcr-9* locus is flanked by two different terminal repeat sequences (IRR) from the IS*5* (orange box) and IS*6* (red box) families. The *mcr-9* upstream region contains highly conserved putative −35 and −10 σ^70^-dependent promoter elements (blue boxes and blue text). Moreover, the *mcr-9* promoter region contains an inverted repeat motif (green box, green text, and sequence logo) that is conserved in more than 95% of 321 *mcr-9* genes, as shown by the sequence logo (constructed using WebLogo) (24).

Three-dimensional (3D) structural models of all nine Mcr homologues (Fig. 2A) based on EptA (2) were constructed using the Phyre2 server (17) and visualized using UCSF Chimera (18). Congruent with the phylogeny based on their amino acid sequences (Fig. 1A), comparisons of different Mcr protein models using Dali (19) revealed that Mcr-3, Mcr-4, Mcr-7, and Mcr-9 were closely related at the structural level (Fig. 1C).

Proteins encoded by *mcr-1* to *-9* revealed high levels of conservation for both the membrane-anchored domain and the soluble catalytic domain (Fig. 2A). Interestingly, analyses of structural models of the nine Mcr homologues using the ESPript 3 server (20) showed that both amino acids and structural elements were conserved on the C-terminal catalytic domain, while only structural elements were conserved on the membrane-anchored N-terminal domain (Fig. 2B).

### Numerous genera of Enterobacteriaceae harbor *mcr-9* on IncHI2 plasmids.

blastp searches of *mcr-9* against NCBI’s nr database revealed that *mcr-9* was present in multiple genera of Enterobacteriaceae (Table S3). The 10 highest-scoring hits in the nr database matched *mcr-9* with at least 99% amino acid identity (including *mcr-9* characterized here [Table S3 and Fig. S1A]); the amino acid identities of the remaining hits with high query coverage (> 90%) dropped below 88% identity (Table S3 and Fig. S1A). *mcr-9* was detected in 335 genomes linked to NCBI identical protein groups (IPGs) associated with the 10 highest-scoring protein accession numbers (accessed 23 January 2019 [see Tables S3 and S6 in the supplemental material]). Analysis of the *mcr-9* promoter region in 321 of these genomes (Text S1) showed conserved putative σ^70^ family-dependent −35 and −10 regions and an inverted repeat (Fig. 2C). The conserved DNA motif in the *mcr-9* promoter is likely a recognition sequence for a transcription regulator, suggesting that additional factors or induction/derepression conditions might be needed for full expression of wild-type *mcr-9*. Promoter variation (21) and testing conditions (22, 23) have been shown to influence *mcr* expression and the colistin MIC, which may explain why the *S.* Typhimurium strain queried here was colistin susceptible under the tested conditions.

10.1128/mBio.00853-19.9 TABLE S6 Location of *mcr-9* on contigs for 335 genome assemblies. Download Table S6, DOCX file, 0.1 MB. Copyright © 2019 Carroll et al. 2019 Carroll et al. This content is distributed under the terms of the Creative Commons Attribution 4.0 International license.

Of the 335 genomes in which *mcr-9* was detected, 65 had at least one plasmid replicon (detected using ABRicate and PlasmidFinder as described above) present on the same contig as *mcr-9*; in 59 of these 65 genomes, IncHI2 and/or IncHI2A replicons were detected on the same contig as *mcr-9* (Table S6). In 32 of the 37 closed genomes in which it was detected, *mcr-9* was harbored on a plasmid (Table S6). These results indicate that *mcr-9* has the potential to reduce susceptibility to colistin, up to and beyond the EUCAST breakpoint, and can be found extrachromosomally in multiple species of Enterobacteriaceae, making it a relevant threat to public health. Future studies querying the plasmids that harbor *mcr-9* (e.g., transferability, stability, and copy number variation) will offer further insight into the potential role that *mcr-9* plays in the dissemination of colistin resistance worldwide.

### Accession number(s).

The nucleotide and amino acid sequences of *mcr-9* are available under NCBI reference sequence accession no. NZ_NAAN01000063.1 (NCBI protein accession no. WP_001572373.1).
