# Supplementary material for: Identification of Novel Mobilized Colistin Resistance Gene mcr-9 in a Multidrug-Resistant, Colistin-Susceptible Salmonella enterica Serotype Typhimurium Isolate
Source: mBio. 2019 May 7;10(3):e00853-19. doi: 10.1128/mBio.00853-19 (PMC6509194; doi:10.1128/mBio.00853-19)
Supplement: TABLE S1 [file mBio.00853-19-st001.docx]

**Supplemental Table S1.** Antimicrobial resistance (AMR) genes and plasmid replicons detected in the assembly of HUM_TYPH_WA_10_R9_3274.^a^

| **Contig^b^** | **Contig Predicted**  **Class (Probability)^c^** | **Feature ID** | **Start** | **End** | **% Coverage** | **% Identity** | **Feature**  **Detection**  **Tool^d^** | **Database^e^** | **Local Alignment**  **Algorithm^f^** |
| --- | --- | --- | --- | --- | --- | --- | --- | --- | --- |
| ***Plasmid Replicons*** |  |  |  |  |  |  |  |  |  |
| NZ_NAAN01000023.1 | Plasmid: Proteobacteria (0.996) | IncFII(S) | 71035 | 71296 | 100 | 100 | ABRicate | PlasmidFinder  (Nucleotide) | blastn |
| NZ_NAAN01000026.1 | Plasmid: Proteobacteria (0.988) | IncHI2A | 21810 | 22439 | 100 | 100 | ABRicate | PlasmidFinder  (Nucleotide) | blastn |
| NZ_NAAN01000026.1 | Plasmid: Proteobacteria (0.988) | TrfA | 36093 | 36687 | 99.66 | 80.37 | ABRicate | PlasmidFinder  (Nucleotide) | blastn |
| NZ_NAAN01000026.1 | Plasmid: Proteobacteria (0.988) | IncHI2 | 37305 | 37631 | 100 | 100 | ABRicate | PlasmidFinder  (Nucleotide) | blastn |
| ***AMR Genes*** |  |  |  |  |  |  |  |  |  |
| NZ_NAAN01000005.1 | Chromosome:  Proteobacteria (0.883) | *aac(6')-Iaa* | 80967 | 81404 | 100 | 100 | ABRicate | ResFinder  (Nucleotide) | blastn |
| NZ_NAAN01000012.1 | Chromosome:  Proteobacteria (0.851) | *aph(3')-Ia* | 122528 | 123343 | 100 | 100 | ABRicate | ResFinder  (Nucleotide) | blastn |
| NZ_NAAN01000043.1 | Plasmid:  Proteobacteria (0.963) | *floR* | 4501 | 5714 | 99.92 | 98.19 | ABRicate | ResFinder  (Nucleotide) | blastn |
| NZ_NAAN01000043.1 | Plasmid:  Proteobacteria (0.963) | *sul2* | 8218 | 9033 | 100 | 100 | ABRicate | ResFinder  (Nucleotide) | blastn |
| NZ_NAAN01000044.1 | Plasmid:  Proteobacteria (0.989) | *aph(3')-Ia* | 1 | 440 | 53.92 | 100 | ABRicate | ResFinder  (Nucleotide) | blastn |
| NZ_NAAN01000044.1 | Plasmid:  Proteobacteria (0.989) | *tet(A)* | 4297 | 5543 | 97.8 | 100 | ABRicate | ResFinder  (Nucleotide) | blastn |
| NZ_NAAN01000046.1 | Plasmid:  Proteobacteria (0.997) | *aac(6')-Iic* | 267 | 848 | 100 | 100 | ABRicate | ResFinder  (Nucleotide) | blastn |
| NZ_NAAN01000046.1 | Plasmid:  Proteobacteria (0.997) | *aac(3)-Iib* | 2933 | 3742 | 99.88 | 78.42 | ABRicate | ResFinder  (Nucleotide) | blastn |
| NZ_NAAN01000046.1 | Plasmid:  Proteobacteria (0.997) | *ere(A)* | 5145 | 6204 | 86.31 | 99.44 | ABRicate | ResFinder  (Nucleotide) | blastn |
| NZ_NAAN01000049.1 | Plasmid:  Proteobacteria (0.997) | *blaTEM-1B* | 149 | 1009 | 100 | 100 | ABRicate | ResFinder  (Nucleotide) | blastn |
| NZ_NAAN01000050.1 | Unclassified:  Proteobacteria (< 0.700) | *blaSHV-12* | 3655 | 4515 | 100 | 100 | ABRicate | ResFinder  (Nucleotide) | blastn |
| NZ_NAAN01000055.1 | Unclassified:  Proteobacteria (< 0.700) | *dfrA18* | 736 | 1305 | 100 | 99.83 | ABRicate | ResFinder  (Nucleotide) | blastn |
| NZ_NAAN01000057.1 | Unclassified:  Proteobacteria (< 0.700) | *tet(D)* | 1769 | 2953 | 100 | 100 | ABRicate | ResFinder  (Nucleotide) | blastn |
| NZ_NAAN01000058.1 | Plasmid:  Proteobacteria (0.953) | *strA* | 262 | 1065 | 100 | 100 | ABRicate | ResFinder  (Nucleotide) | blastn |
| NZ_NAAN01000058.1 | Plasmid:  Proteobacteria (0.953) | *aph(6)-Id* | 1065 | 1901 | 100 | 100 | ABRicate | ResFinder  (Nucleotide) | blastn |
| NZ_NAAN01000062.1 | Plasmid:  Proteobacteria (0.792) | *qnrB2* | 420 | 1064 | 100 | 100 | ABRicate | ResFinder  (Nucleotide) | blastn |
| NZ_NAAN01000063.1 | Chromosome:  Bacteroidetes (0.713) | *mcr-*3.17 | 789^g^ | 2402 | 99.45 | 64.50 | BTyper | ResFinder + Transeq  (Amino Acid) | tblastn |
| NZ_NAAN01000072.1 | Unclassified:  Proteobacteria (< 0.700) | *ant(3'')-Ia* | 180 | 1147 | 99.59 | 99.59 | ABRicate | ResFinder  (Nucleotide) | blastn |
| NZ_NAAN01000073.1 | Plasmid:  Proteobacteria (0.965) | *aadA2* | 229 | 1041 | 99.27 | 99.88 | ABRicate | ResFinder  (Nucleotide) | blastn |
| NZ_NAAN01000076.1 | Plasmid:  Proteobacteria (0.992) | *sul1* | 172 | 1038 | 100 | 99.89 | ABRicate | ResFinder  (Nucleotide) | blastn |
| NZ_NAAN01000091.1 | Not applicable^h^ | *aph(3')-Ia* | 1 | 503 | 61.64 | 100 | ABRicate | ResFinder  (Nucleotide) | blastn |

^a^NCBI RefSeq Accession GCF_002091095.1

^b^Refers to NCBI Reference Sequence accession numbers for contigs in the assembly

^c^One of 26 plasmid or chromosomal classes to which each contig was assigned using PlasFlow version 1.0 (P.S. Krawczyk, L. Lipinski, and A. Dziembowski, Nucleic Acids Res 46(6):e35, 2018, doi: 10.1093/nar/gkx1321), with the corresponding assignment probability in parentheses; if the assignment probability was lower than 0.7 (< 0.700), the contig was considered unclassified

^d^*In silico* tool used to detect feature; either ABRicate (ABRicate version 0.8; https://github.com/tseemann/abricate) or BTyper (BTyper version 2.3.2; L.M. Carroll, J. Kovac, R.A. Miller, and M. Wiedmann, Appl Environ Microbiol 83(17): e01096-17, 2017, doi: 10.1128/AEM.01096-17)

^e^Database used to detect feature; either PlasmidFinder (nucleotide sequences, accessed June 11, 2018; A. Carattoli, et al., Antimicrob Agents Chemother 58(7): 3895-3903, 2014, doi: 10.1128/AAC.02412-14), ResFinder (nucleotide sequences, accessed June 11, 2018; E. Zankari, et al., J Antimicrob Chemother 67(11): 2640-2644, 2012, doi: 10.1093/jac/dks261), or ResFinder + Transeq (amino acid sequences; ResFinder nucleotide sequences accessed January 22, 2019, translated using EMBOSS Transeq in reading frame 1 [https://www.ebi.ac.uk/Tools/st/emboss_transeq/])

^f^Algorithm used by the respective feature detection tool/database combination to detect feature; either blastn (nucleotide blast with minimum identity thresholds of 80 and 75% and minimum coverage thresholds of 60 and 50%, for PlasmidFinder and ResFinder, respectively) or tblastn (translated nucleotide blast with minimum identity and coverage thresholds of 50 and 70%, respectively) (C. Camacho, et al., BMC Bioinformatics 10:421, 2009, doi: 10.1186/1471-2105-10-421)

^g^Reported as 789 by BTyper and as 783 in NCBI Reference Sequence NZ_NAAN01000063.1

^h^Contig NZ_NAAN01000091.1 was not classified using PlasFlow due to its small size (< 1000 bp)
