# Supplementary material for: Identification of Novel Mobilized Colistin Resistance Gene mcr-9 in a Multidrug-Resistant, Colistin-Susceptible Salmonella enterica Serotype Typhimurium Isolate
Source: mBio. 2019 May 7;10(3):e00853-19. doi: 10.1128/mBio.00853-19 (PMC6509194; doi:10.1128/mBio.00853-19)
Supplement: TABLE S2 [file mBio.00853-19-st002.docx]

**Supplemental Table S2.** Comparison of *mcr*-9 to previously published *mcr* homologues using protein blast (blastp).^a^

| **Query Sequence ID^b^** | **Subject Sequence ID^c^** | **Percent (%) Identity** | **Alignment Length** | **Bit Score** | **E-Value** | **Query Coverage**  **per Subject (%)** |
| --- | --- | --- | --- | --- | --- | --- |
| WP_001572373.1 | mcr-3.6_1_MF598076_1 | 65.8 | 538 | 784 | 0 | 99 |
| WP_001572373.1 | mcr-3.16_1_MH332766_1 | 65.6 | 538 | 783 | 0 | 99 |
| WP_001572373.1 | mcr-3.15_1_MH332765_1 | 65.4 | 538 | 782 | 0 | 99 |
| WP_001572373.1 | mcr-3.8_1_MF598078_1 | 65.6 | 538 | 781 | 0 | 99 |
| WP_001572373.1 | mcr-3.3_1_MF495680_1 | 65.6 | 538 | 781 | 0 | 99 |
| WP_001572373.1 | mcr-3.18_1_MH332768_1 | 65.4 | 538 | 780 | 0 | 99 |
| WP_001572373.1 | mcr-3.5_1_MF489760_1 | 65.1 | 538 | 780 | 0 | 99 |
| WP_001572373.1 | mcr-3.25_1_NG060585_1 | 65.4 | 538 | 779 | 0 | 99 |
| WP_001572373.1 | mcr-3.2_1_NMWW01000143_1 | 64.9 | 538 | 778 | 0 | 99 |
| WP_001572373.1 | mcr-3.13_1_MH332763_1 | 65.2 | 538 | 778 | 0 | 99 |
| WP_001572373.1 | mcr-3.21_1_NG060582_1 | 64.9 | 538 | 778 | 0 | 99 |
| WP_001572373.1 | mcr-3.20_1_NG055493_1 | 64.7 | 538 | 777 | 0 | 99 |
| WP_001572373.1 | mcr-3.1_1_KY924928_1 | 64.7 | 538 | 777 | 0 | 99 |
| WP_001572373.1 | mcr-3.22_1_NG060581_1 | 64.7 | 538 | 776 | 0 | 99 |
| WP_001572373.1 | mcr-3.19_1_NG055497_1 | 64.7 | 538 | 776 | 0 | 99 |
| WP_001572373.1 | mcr-3.17_1_MH332767_1 | 64.5 | 538 | 776 | 0 | 99 |
| WP_001572373.1 | mcr-3.4_1_FLXA01000011_1 | 64.7 | 538 | 776 | 0 | 99 |
| WP_001572373.1 | mcr-3.24_1_NG060580_1 | 64.7 | 538 | 776 | 0 | 99 |
| WP_001572373.1 | mcr-3.11_1_MG489958_1 | 64.7 | 538 | 776 | 0 | 99 |
| WP_001572373.1 | mcr-3.12_1_MG564491_1 | 65.2 | 538 | 775 | 0 | 99 |
| WP_001572373.1 | mcr-3.23_1_NG060583_1 | 64.5 | 538 | 775 | 0 | 99 |
| WP_001572373.1 | mcr-3.7_1_MF598077_1 | 65.2 | 538 | 775 | 0 | 99 |
| WP_001572373.1 | mcr-3.10_1_MG214531_1 | 65.2 | 538 | 774 | 0 | 99 |
| WP_001572373.1 | mcr-3.14_1_MH332764_1 | 65.1 | 538 | 774 | 0 | 99 |
| WP_001572373.1 | mcr-3.9_1_MF598080_1 | 65.1 | 538 | 773 | 0 | 99 |
| WP_001572373.1 | mcr-7.1_1_MG267386_1 | 63.0 | 514 | 740 | 0 | 95 |
| WP_001572373.1 | mcr-4.3_1_MG026621_1 | 43.4 | 535 | 489 | 1.20E-171 | 99 |
| WP_001572373.1 | mcr-4.4_1_MG822665_1 | 43.2 | 542 | 488 | 1.92E-171 | 99 |
| WP_001572373.1 | mcr-4.2_1_MG822663_1 | 43.6 | 530 | 488 | 3.76E-171 | 98 |
| WP_001572373.1 | mcr-4.1_1_MF543359_1 | 43.0 | 542 | 486 | 1.96E-170 | 99 |
| WP_001572373.1 | mcr-4.6_1_MH423812_1 | 43.0 | 542 | 485 | 3.42E-170 | 99 |
| WP_001572373.1 | mcr-4.5_1_MG822664_1 | 43.0 | 542 | 484 | 1.42E-169 | 99 |
| WP_001572373.1 | mcr-8_1_MG736312_1 | 44.8 | 498 | 451 | 1.87E-156 | 91 |
| WP_001572373.1 | mcr-5.2_1_MG384740_1 | 33.3 | 553 | 337 | 2.18E-112 | 99 |
| WP_001572373.1 | mcr-5.1_1_KY807921_1 | 33.2 | 554 | 337 | 3.30E-112 | 99 |
| WP_001572373.1 | mcr-1.5_1_KY283125_1 | 36.4 | 535 | 327 | 1.96E-108 | 97 |
| WP_001572373.1 | mcr-1.9_1_KY964067_1 | 36.3 | 535 | 326 | 4.93E-108 | 97 |
| WP_001572373.1 | mcr-1.4_1_KY041856_1 | 36.3 | 535 | 325 | 6.73E-108 | 97 |
| WP_001572373.1 | mcr-1.2_1_KX236309_1 | 36.3 | 535 | 325 | 8.43E-108 | 97 |
| WP_001572373.1 | mcr-1.8_1_KY683842_1 | 36.3 | 535 | 325 | 8.99E-108 | 97 |
| WP_001572373.1 | mcr-1.1_1_KP347127_1 | 36.3 | 535 | 325 | 9.69E-108 | 97 |
| WP_001572373.1 | mcr-1.13_1_MG384739_1 | 36.3 | 535 | 325 | 1.02E-107 | 97 |
| WP_001572373.1 | mcr-1.11_1_KY853650_1 | 36.3 | 535 | 325 | 1.05E-107 | 97 |
| WP_001572373.1 | mcr-1.12_1_LC337668_1 | 36.3 | 535 | 325 | 1.07E-107 | 97 |
| WP_001572373.1 | mcr-1.7_1_KY488488_1 | 36.3 | 535 | 325 | 1.19E-107 | 97 |
| WP_001572373.1 | mcr-1.6_1_KY352406_1 | 36.3 | 535 | 325 | 1.21E-107 | 97 |
| WP_001572373.1 | mcr-1.3_1_KU934208_1 | 36.3 | 535 | 325 | 1.29E-107 | 97 |
| WP_001572373.1 | mcr-1.10_1_MF176238_1 | 35.9 | 535 | 323 | 5.74E-107 | 97 |
| WP_001572373.1 | mcr-1.14_1_LS398440_1 | 36.3 | 535 | 322 | 2.34E-106 | 97 |
| WP_001572373.1 | mcr-2.2_1_MF176239_1 | 34.3 | 534 | 312 | 7.12E-103 | 96 |
| WP_001572373.1 | mcr-2.1_1_LT598652_1 | 33.9 | 534 | 310 | 5.92E-102 | 96 |
| WP_001572373.1 | mcr-6.1_1_MF176240_1 | 33.7 | 525 | 296 | 1.70E-96 | 94 |

^a^The 52 *mcr* nucleotide sequences available in ResFinder (accessed January 22, 2019; E. Zankari, et al., J Antimicrob Chemother 67(11): 2640-2644, 2012, doi: 10.1093/jac/dks261) were translated into amino acid sequences using EMBOSS Transeq (reading frame 1; https://www.ebi.ac.uk/Tools/st/emboss_transeq/), and the resulting amino acid sequences were used as a database to which *mcr*-9 was aligned using the command-line implementation of protein blast (blastp) version 2.7.1 and default alignment and scoring parameters (C. Camacho, et al., BMC Bioinformatics 10:421, 2009, doi: 10.1186/1471-2105-10-421).

^b^Refers to *mcr-*9 (NCBI Protein Accession WP_001572373.1), which was treated as the query for blastp.

^c^Refers to one of 52 *mcr* genes available in ResFinder, which was used as the database for blastp.
