# Supplementary material for: Identification of Novel Mobilized Colistin Resistance Gene mcr-9 in a Multidrug-Resistant, Colistin-Susceptible Salmonella enterica Serotype Typhimurium Isolate
Source: mBio. 2019 May 7;10(3):e00853-19. doi: 10.1128/mBio.00853-19 (PMC6509194; doi:10.1128/mBio.00853-19)
Supplement: TABLE S3 [file mBio.00853-19-st003.docx]

**Supplemental Table S3.** Top 100 hits obtained by querying *mcr*-9 against NCBI’s non-redundant protein sequence (nr) database using protein blast (blastp)^a^

| **Subject Sequence ID^b^** | **NCBI Sequence ID and Description^c^** | **Percent (%) Identity** | **Alignment Length** | **Bit Score** | **E-Value** | **Query Coverage per Subject (%)** |
| --- | --- | --- | --- | --- | --- | --- |
| Enterobacteriaceae_WP_044704969.1 | WP_044704969.1 MULTISPECIES: phosphoethanolamine--lipid A transferase, partial [Enterobacteriaceae] | 100 | 539 | 1125 | 0 | 100 |
| **mcr-9_Salmonella_enterica_WP_001572373.1** | **WP_001572373.1 MULTISPECIES: phosphoethanolamine--lipid A transferase [Enterobacterales]** | **100** | **539** | **1125** | **0** | **100** |
| Enterobacteriaceae_WP_012477388.1 | WP_012477388.1 MULTISPECIES: phosphoethanolamine--lipid A transferase [Enterobacteriaceae] | 100 | 539 | 1125 | 0 | 100 |
| Salmonella_enterica_WP_053533432.1 | WP_053533432.1 phosphoethanolamine--lipid A transferase [Salmonella enterica] | 99.814 | 539 | 1124 | 0 | 100 |
| Enterobacter_cloacae_WP_101429035.1 | WP_101429035.1 phosphoethanolamine--lipid A transferase [Enterobacter cloacae] | 99.814 | 539 | 1123 | 0 | 100 |
| Serratia_marcescens_WP_101456540.1 | WP_101456540.1 phosphoethanolamine--lipid A transferase [Serratia marcescens] | 99.814 | 539 | 1122 | 0 | 100 |
| Enterobacter_cloacae_WP_100167873.1 | WP_100167873.1 phosphoethanolamine--lipid A transferase [Enterobacter cloacae] | 99.814 | 539 | 1122 | 0 | 100 |
| Enterobacter_kobei_WP_111969906.1 | WP_111969906.1 phosphoethanolamine--lipid A transferase [Enterobacter kobei] | 99.629 | 539 | 1117 | 0 | 100 |
| Enterobacter_cloacae_WP_063932934.1 | WP_063932934.1 phosphoethanolamine--lipid A transferase, partial [Enterobacter cloacae] | 100 | 521 | 1085 | 0 | 97 |
| Escherichia_coli_R178_CDI45279.1 | CDI45279.1 hypothetical protein [Escherichia coli R178] | 100 | 508 | 1063 | 0 | 94 |
| Buttiauxella_brennerae_WP_064558897.1 | WP_064558897.1 phosphoethanolamine--lipid A transferase [Buttiauxella brennerae] | 86.827 | 539 | 1011 | 0 | 100 |
| Buttiauxella_gaviniae_WP_064511805.1 | WP_064511805.1 phosphoethanolamine--lipid A transferase [Buttiauxella gaviniae] | 86.642 | 539 | 1009 | 0 | 100 |
| Enterobacter_cloacae_SAH12939.1 | SAH12939.1 putative cell division protein [Enterobacter cloacae] | 100 | 477 | 1003 | 0 | 88 |
| Buttiauxella_noackiae_WP_034455328.1 | WP_034455328.1 phosphoethanolamine--lipid A transferase [Buttiauxella noackiae] | 85.316 | 538 | 998 | 0 | 99 |
| Buttiauxella_noackiae_ATCC_51607_OAT18713.1 | OAT18713.1 lipid A 1-phosphate phosphoethanolamine transferase [Buttiauxella noackiae ATCC 51607] | 85.13 | 538 | 978 | 0 | 99 |
| Buttiauxella_noackiae_WP_064554336.1 | WP_064554336.1 phosphoethanolamine--lipid A transferase [Buttiauxella noackiae] | 85.13 | 538 | 978 | 0 | 99 |
| Buttiauxella_sp._3AFRM03_WP_121813602.1 | WP_121813602.1 phosphoethanolamine--lipid A transferase [Buttiauxella sp. 3AFRM03] | 84.758 | 538 | 974 | 0 | 99 |
| Buttiauxella_ferragutiae_WP_064546189.1 | WP_064546189.1 phosphoethanolamine--lipid A transferase [Buttiauxella ferragutiae] | 84.572 | 538 | 972 | 0 | 99 |
| Buttiauxella_agrestis_WP_115628344.1 | WP_115628344.1 phosphoethanolamine--lipid A transferase [Buttiauxella agrestis] | 80.669 | 538 | 960 | 0 | 99 |
| Buttiauxella_agrestis_WP_034495833.1 | WP_034495833.1 phosphoethanolamine--lipid A transferase [Buttiauxella agrestis] | 80.297 | 538 | 953 | 0 | 99 |
| Enterobacter_roggenkampii_WP_059359330.1 | WP_059359330.1 phosphoethanolamine--lipid A transferase [Enterobacter roggenkampii] | 82.931 | 539 | 943 | 0 | 100 |
| Enterobacteriaceae_WP_023332837.1 | WP_023332837.1 MULTISPECIES: phosphoethanolamine--lipid A transferase [Enterobacteriaceae] | 82.931 | 539 | 943 | 0 | 100 |
| Enterobacter_hormaechei_WP_111976222.1 | WP_111976222.1 phosphoethanolamine--lipid A transferase, partial [Enterobacter hormaechei] | 100 | 444 | 937 | 0 | 82 |
| Enterobacter_sp._MGH_14_ERO99995.1 | ERO99995.1 hypothetical protein L360_04927 [Enterobacter sp. MGH 14] | 100 | 440 | 927 | 0 | 82 |
| Buttiauxella_izardii_WP_120062886.1 | WP_120062886.1 phosphoethanolamine--lipid A transferase [Buttiauxella izardii] | 81.97 | 538 | 925 | 0 | 99 |
| Escherichia_coli_KUH14423.1 | KUH14423.1 hydrolase, partial [Escherichia coli] | 82.966 | 499 | 897 | 0 | 93 |
| Escherichia_coli_WP_077871934.1 | WP_077871934.1 phosphoethanolamine--lipid A transferase [Escherichia coli] | 82.731 | 498 | 894 | 0 | 92 |
| Shimwellia_blattae_WP_034920248.1 | WP_034920248.1 phosphoethanolamine--lipid A transferase [Shimwellia blattae] | 73.098 | 539 | 878 | 0 | 100 |
| Shimwellia_blattae_DSM_4481_=_NBRC_105725_AFJ46246.1 | AFJ46246.1 hypothetical protein EBL_c11420 [Shimwellia blattae DSM 4481 = NBRC 105725] | 73.098 | 539 | 878 | 0 | 100 |
| Shimwellia_blattae_DSM_4481_=_NBRC_105725_GAB81118.1 | GAB81118.1 phosphoethanolamine transferase EptA [Shimwellia blattae DSM 4481 = NBRC 105725] | 72.727 | 528 | 857 | 0 | 98 |
| Aeromonas_sobria_WP_101324982.1 | WP_101324982.1 MCR-3-related phosphoethanolamine--lipid A transferase [Aeromonas sobria] | 67.355 | 533 | 813 | 0 | 99 |
| Aeromonas_WP_103828101.1 | WP_103828101.1 MULTISPECIES: MCR-3-related phosphoethanolamine--lipid A transferase [Aeromonas] | 65.863 | 539 | 808 | 0 | 100 |
| Aeromonas_WP_108541360.1 | WP_108541360.1 MULTISPECIES: MCR-3-related phosphoethanolamine--lipid A transferase [Aeromonas] | 66.979 | 533 | 807 | 0 | 99 |
| Aeromonas_WP_043169858.1 | WP_043169858.1 MULTISPECIES: MCR-3-related phosphoethanolamine--lipid A transferase [Aeromonas] | 66.357 | 538 | 806 | 0 | 99 |
| Aeromonas_salmonicida_WP_005321524.1 | WP_005321524.1 MCR-3-related phosphoethanolamine--lipid A transferase [Aeromonas salmonicida] | 66.604 | 533 | 806 | 0 | 99 |
| Aeromonas_dhakensis_WP_042049508.1 | WP_042049508.1 MCR-3-related phosphoethanolamine--lipid A transferase [Aeromonas dhakensis] | 66.357 | 538 | 805 | 0 | 99 |
| Aeromonas_hydrophila_WP_073350735.1 | WP_073350735.1 MCR-3-related phosphoethanolamine--lipid A transferase [Aeromonas hydrophila] | 65.799 | 538 | 805 | 0 | 99 |
| Aeromonas_dhakensis_WP_017778796.1 | WP_017778796.1 MCR-3-related phosphoethanolamine--lipid A transferase [Aeromonas dhakensis] | 66.171 | 538 | 805 | 0 | 99 |
| Aeromonas_hydrophila_WP_029302738.1 | WP_029302738.1 MCR-3-related phosphoethanolamine--lipid A transferase [Aeromonas hydrophila] | 65.799 | 538 | 805 | 0 | 99 |
| Aeromonas_sp._EERV15_WP_068977109.1 | WP_068977109.1 MCR-3-related phosphoethanolamine--lipid A transferase [Aeromonas sp. EERV15] | 66.792 | 533 | 805 | 0 | 99 |
| Aeromonas_hydrophila_WP_118880936.1 | WP_118880936.1 MCR-3-related phosphoethanolamine--lipid A transferase [Aeromonas hydrophila] | 65.799 | 538 | 805 | 0 | 99 |
| Aeromonas_hydrophila_WP_060390254.1 | WP_060390254.1 MCR-3-related phosphoethanolamine--lipid A transferase [Aeromonas hydrophila] | 65.799 | 538 | 805 | 0 | 99 |
| Aeromonas_hydrophila_WP_025201501.1 | WP_025201501.1 MCR-3-related phosphoethanolamine--lipid A transferase [Aeromonas hydrophila] | 66.171 | 538 | 805 | 0 | 99 |
| Aeromonas_hydrophila_WP_043162165.1 | WP_043162165.1 MCR-3-related phosphoethanolamine--lipid A transferase [Aeromonas hydrophila] | 65.613 | 538 | 805 | 0 | 99 |
| Aeromonas_hydrophila_WP_113994538.1 | WP_113994538.1 MCR-3-related phosphoethanolamine--lipid A transferase [Aeromonas hydrophila] | 65.799 | 538 | 805 | 0 | 99 |
| Aeromonas_hydrophila_WP_024944509.1 | WP_024944509.1 MCR-3-related phosphoethanolamine--lipid A transferase [Aeromonas hydrophila] | 65.613 | 538 | 805 | 0 | 99 |
| Aeromonas_hydrophila_WP_017408045.1 | WP_017408045.1 MCR-3-related phosphoethanolamine--lipid A transferase [Aeromonas hydrophila] | 65.613 | 538 | 804 | 0 | 99 |
| Aeromonas_WP_017764183.1 | WP_017764183.1 MULTISPECIES: MCR-3-related phosphoethanolamine--lipid A transferase [Aeromonas] | 65.985 | 538 | 804 | 0 | 99 |
| Aeromonas_dhakensis_WP_005302376.1 | WP_005302376.1 MCR-3-related phosphoethanolamine--lipid A transferase [Aeromonas dhakensis] | 65.985 | 538 | 804 | 0 | 99 |
| Aeromonas_dhakensis_WP_042888944.1 | WP_042888944.1 MCR-3-related phosphoethanolamine--lipid A transferase [Aeromonas dhakensis] | 65.985 | 538 | 804 | 0 | 99 |
| Aeromonas_hydrophila_WP_049049373.1 | WP_049049373.1 MCR-3-related phosphoethanolamine--lipid A transferase [Aeromonas hydrophila] | 65.613 | 538 | 804 | 0 | 99 |
| Aeromonas_hydrophila_WP_101150900.1 | WP_101150900.1 MCR-3-related phosphoethanolamine--lipid A transferase [Aeromonas hydrophila] | 65.799 | 538 | 804 | 0 | 99 |
| Aeromonas_hydrophila_WP_011706485.1 | WP_011706485.1 MCR-3-related phosphoethanolamine--lipid A transferase [Aeromonas hydrophila] | 65.799 | 538 | 804 | 0 | 99 |
| Aeromonas_hydrophila_WP_024941089.1 | WP_024941089.1 MCR-3-related phosphoethanolamine--lipid A transferase [Aeromonas hydrophila] | 65.985 | 538 | 803 | 0 | 99 |
| Aeromonas_hydrophila_WP_029300214.1 | WP_029300214.1 MCR-3-related phosphoethanolamine--lipid A transferase [Aeromonas hydrophila] | 65.613 | 538 | 803 | 0 | 99 |
| Aeromonas_hydrophila_WP_039213234.1 | WP_039213234.1 MCR-3-related phosphoethanolamine--lipid A transferase [Aeromonas hydrophila] | 65.613 | 538 | 803 | 0 | 99 |
| Aeromonas_hydrophila_WP_044800259.1 | WP_044800259.1 MCR-3-related phosphoethanolamine--lipid A transferase [Aeromonas hydrophila] | 65.613 | 538 | 803 | 0 | 99 |
| Aeromonas_hydrophila_WP_101615329.1 | WP_101615329.1 MCR-3-related phosphoethanolamine--lipid A transferase [Aeromonas hydrophila] | 65.613 | 538 | 803 | 0 | 99 |
| Aeromonas_dhakensis_WP_124250425.1 | WP_124250425.1 MCR-3-related phosphoethanolamine--lipid A transferase [Aeromonas dhakensis] | 65.985 | 538 | 803 | 0 | 99 |
| Aeromonas_hydrophila_WP_045789498.1 | WP_045789498.1 MCR-3-related phosphoethanolamine--lipid A transferase [Aeromonas hydrophila] | 65.428 | 538 | 803 | 0 | 99 |
| Aeromonas_hydrophila_WP_043123781.1 | WP_043123781.1 MCR-3-related phosphoethanolamine--lipid A transferase [Aeromonas hydrophila] | 65.428 | 538 | 803 | 0 | 99 |
| Aeromonas_dhakensis_WP_054544648.1 | WP_054544648.1 MCR-3-related phosphoethanolamine--lipid A transferase [Aeromonas dhakensis] | 65.985 | 538 | 803 | 0 | 99 |
| Aeromonas_dhakensis_WP_017786737.1 | WP_017786737.1 MCR-3-related phosphoethanolamine--lipid A transferase [Aeromonas dhakensis] | 65.799 | 538 | 802 | 0 | 99 |
| Aeromonas_hydrophila_WP_124251534.1 | WP_124251534.1 MCR-3-related phosphoethanolamine--lipid A transferase [Aeromonas hydrophila] | 65.428 | 538 | 802 | 0 | 99 |
| Aeromonas_dhakensis_WP_065017960.1 | WP_065017960.1 MCR-3-related phosphoethanolamine--lipid A transferase [Aeromonas dhakensis] | 65.799 | 538 | 802 | 0 | 99 |
| Aeromonas_dhakensis_WP_026459105.1 | WP_026459105.1 MCR-3-related phosphoethanolamine--lipid A transferase [Aeromonas dhakensis] | 65.799 | 538 | 802 | 0 | 99 |
| Aeromonas_hydrophila_WP_106552789.1 | WP_106552789.1 MCR-3-related phosphoethanolamine--lipid A transferase [Aeromonas hydrophila] | 65.492 | 539 | 802 | 0 | 100 |
| Aeromonas_hydrophila_WP_017783002.1 | WP_017783002.1 MCR-3-related phosphoethanolamine--lipid A transferase [Aeromonas hydrophila] | 65.613 | 538 | 801 | 0 | 99 |
| Aeromonas_hydrophila_WP_102988495.1 | WP_102988495.1 MCR-3-related phosphoethanolamine--lipid A transferase [Aeromonas hydrophila] | 65.428 | 538 | 801 | 0 | 99 |
| Aeromonas_hydrophila_WP_043119141.1 | WP_043119141.1 MCR-3-related phosphoethanolamine--lipid A transferase [Aeromonas hydrophila] | 65.613 | 538 | 801 | 0 | 99 |
| Aeromonas_WP_076361029.1 | WP_076361029.1 MULTISPECIES: MCR-3-related phosphoethanolamine--lipid A transferase [Aeromonas] | 65.613 | 538 | 801 | 0 | 99 |
| Aeromonas_hydrophila_WP_042066724.1 | WP_042066724.1 MCR-3-related phosphoethanolamine--lipid A transferase [Aeromonas hydrophila] | 65.428 | 538 | 800 | 0 | 99 |
| Aeromonas_hydrophila_WP_041217604.1 | WP_041217604.1 MCR-3-related phosphoethanolamine--lipid A transferase [Aeromonas hydrophila] | 65.428 | 538 | 800 | 0 | 99 |
| Aeromonas_hydrophila_WP_077097776.1 | WP_077097776.1 MCR-3-related phosphoethanolamine--lipid A transferase [Aeromonas hydrophila] | 65.242 | 538 | 800 | 0 | 99 |
| Aeromonas_hydrophila_WP_043164956.1 | WP_043164956.1 MCR-3-related phosphoethanolamine--lipid A transferase [Aeromonas hydrophila] | 65.428 | 538 | 800 | 0 | 99 |
| Aeromonas_dhakensis_WP_123246414.1 | WP_123246414.1 MCR-3-related phosphoethanolamine--lipid A transferase [Aeromonas dhakensis] | 65.799 | 538 | 800 | 0 | 99 |
| Aeromonas_piscicola_WP_042867079.1 | WP_042867079.1 MCR-3-related phosphoethanolamine--lipid A transferase [Aeromonas piscicola] | 65.428 | 538 | 794 | 0 | 99 |
| Aeromonas_allosaccharophila_WP_111809541.1 | WP_111809541.1 MCR-3-related phosphoethanolamine--lipid A transferase [Aeromonas allosaccharophila] | 66.357 | 538 | 792 | 0 | 99 |
| Salmonella_enterica_WP_080229728.1 | WP_080229728.1 DUF1705 domain-containing protein, partial [Salmonella enterica] | 100 | 383 | 790 | 0 | 71 |
| Aeromonas_WP_042649073.1 | WP_042649073.1 MULTISPECIES: MCR-3-related phosphoethanolamine--lipid A transferase [Aeromonas] | 67.917 | 533 | 790 | 0 | 99 |
| Aeromonas_caviae_WP_039039920.1 | WP_039039920.1 MCR-3-related phosphoethanolamine--lipid A transferase [Aeromonas caviae] | 67.355 | 533 | 789 | 0 | 99 |
| Aeromonas_veronii_WP_118854325.1 | WP_118854325.1 MCR-3-related phosphoethanolamine--lipid A transferase [Aeromonas veronii] | 67.73 | 533 | 789 | 0 | 99 |
| Aeromonas_sp._ASNIH5_WP_103243482.1 | WP_103243482.1 phosphoethanolamine--lipid A transferase [Aeromonas sp. ASNIH5] | 66.417 | 533 | 789 | 0 | 99 |
| Aeromonas_jandaei_WP_124242181.1 | WP_124242181.1 MCR-3-related phosphoethanolamine--lipid A transferase [Aeromonas jandaei] | 67.542 | 533 | 788 | 0 | 99 |
| Aeromonas_WP_017778763.1 | WP_017778763.1 MULTISPECIES: MCR-3-related phosphoethanolamine--lipid A transferase [Aeromonas] | 67.355 | 533 | 788 | 0 | 99 |
| Aeromonas_sp._YN13HZO-058_WP_075114467.1 | WP_075114467.1 MCR-3-related phosphoethanolamine--lipid A transferase [Aeromonas sp. YN13HZO-058] | 66.357 | 538 | 788 | 0 | 99 |
| Aeromonas_WP_101531736.1 | WP_101531736.1 MULTISPECIES: MCR-3-related phosphoethanolamine--lipid A transferase [Aeromonas] | 67.542 | 533 | 787 | 0 | 99 |
| Aeromonas_veronii_WP_121463520.1 | WP_121463520.1 MCR-3-related phosphoethanolamine--lipid A transferase [Aeromonas veronii] | 67.542 | 533 | 787 | 0 | 99 |
| Aeromonas_jandaei_WP_042032311.1 | WP_042032311.1 MCR-3-related phosphoethanolamine--lipid A transferase [Aeromonas jandaei] | 66.355 | 535 | 786 | 0 | 99 |
| Aeromonas_sp._EERV15_WP_068976857.1 | WP_068976857.1 MCR-3-related phosphoethanolamine--lipid A transferase [Aeromonas sp. EERV15] | 66.419 | 539 | 786 | 0 | 99 |
| Aeromonas_veronii_WP_118854731.1 | WP_118854731.1 MCR-3-related phosphoethanolamine--lipid A transferase [Aeromonas veronii] | 67.355 | 533 | 786 | 0 | 99 |
| Aeromonas_WP_042649074.1 | WP_042649074.1 MULTISPECIES: phosphoethanolamine--lipid A transferase MCR-3.6 [Aeromonas] | 65.799 | 538 | 785 | 0 | 99 |
| Aeromonas_sp._SCS5_WP_083593337.1 | WP_083593337.1 MCR-3-related phosphoethanolamine--lipid A transferase [Aeromonas sp. SCS5] | 66.229 | 533 | 785 | 0 | 99 |
| Aeromonas_jandaei_WP_104014612.1 | WP_104014612.1 MCR-3-related phosphoethanolamine--lipid A transferase [Aeromonas jandaei] | 66.168 | 535 | 784 | 0 | 99 |
| Aeromonas_sp._CU5_WP_098968686.1 | WP_098968686.1 MCR-3-related phosphoethanolamine--lipid A transferase [Aeromonas sp. CU5] | 66.419 | 539 | 784 | 0 | 99 |
| Aeromonas_hydrophila_WP_017784890.1 | WP_017784890.1 MCR-3-related phosphoethanolamine--lipid A transferase [Aeromonas hydrophila] | 66.171 | 538 | 784 | 0 | 99 |
| Aeromonas_hydrophila_ML09-119_AGM44602.1 | AGM44602.1 sulfatase [Aeromonas hydrophila ML09-119] | 66.221 | 524 | 783 | 0 | 97 |
| Aeromonas_jandaei_WP_041207471.1 | WP_041207471.1 MCR-3-related phosphoethanolamine--lipid A transferase [Aeromonas jandaei] | 65.794 | 535 | 783 | 0 | 99 |
| Aeromonas_veronii_ASU10318.1 | ASU10318.1 phosphoethanolamine transferase [Aeromonas veronii] | 67.355 | 533 | 783 | 0 | 99 |
| Aeromonas_lacus_WP_084059638.1 | WP_084059638.1 MCR-3-related phosphoethanolamine--lipid A transferase [Aeromonas lacus] | 66.229 | 533 | 783 | 0 | 99 |

^a^The amino acid sequence of *mcr*-9 (NCBI Protein Accession WP_001572373.1) was queried against NCBI’s non-redundant protein sequence (nr) database using the protein blast (blastp) webserver (https://blast.ncbi.nlm.nih.gov/Blast.cgi?PAGE=Proteins; accessed January 22, 2019) using default parameters.

^b^Refers to the identifier used in this study (taxonomic information, followed by NCBI Protein Accession number); the entry in boldfaced text refers to *mcr*-9 itself, as it was present in the nr database and matched with the *mcr*-9 query (to avoid redundancy, it has been omitted from phylogenies constructed using this data set)

^c^Refers to NCBI nr database Protein Accession number and description
