# Supplementary material for: Identification of Novel Mobilized Colistin Resistance Gene mcr-9 in a Multidrug-Resistant, Colistin-Susceptible Salmonella enterica Serotype Typhimurium Isolate
Source: mBio. 2019 May 7;10(3):e00853-19. doi: 10.1128/mBio.00853-19 (PMC6509194; doi:10.1128/mBio.00853-19)
Supplement: TABLE S4 [file mBio.00853-19-st004.docx]

**Supplemental Table S4.** Comparison of *mcr*-9 to putative phosphoethanolamine transferases used in other papers^a^ describing novel *mcr* genes.^b^

| **Query Sequence ID^c^** | **Subject Sequence ID^d^** | **Percent (%) Identity** | **Alignment Length** | **Bit Score** | **E-Value** | **Query Coverage per Subject (%)** |
| --- | --- | --- | --- | --- | --- | --- |
| WP_001572373.1 | Aeromonas_hydrophila_KGY49534.1 | 65.428 | 538 | 802 | 0 | 99 |
| WP_001572373.1 | Aeromonas_lacus_WP_033115156.1 | 65.981 | 535 | 783 | 0 | 99 |
| WP_001572373.1 | Aeromonas_dhakensis_WP_017778762.1 | 65.242 | 538 | 781 | 0 | 99 |
| WP_001572373.1 | Aeromonas_caviae_WP_039039919.1 | 65.056 | 538 | 781 | 0 | 99 |
| WP_001572373.1 | Aeromonas_bestiarum_WP_043556478.1 | 64.312 | 538 | 780 | 0 | 99 |
| WP_001572373.1 | Aeromonas_sp._L_1B5_3_KIQ80141.1 | 65.607 | 535 | 779 | 0 | 99 |
| WP_001572373.1 | Aeromonas_sobria_WP_042021679.1 | 66.048 | 539 | 779 | 0 | 99 |
| WP_001572373.1 | Klebsiella_WP_094312656.1 | 64.87 | 538 | 778 | 0 | 99 |
| WP_001572373.1 | Salmonella_enterica_subsp._enterica_serovar_Typhimurium_ORG07507.1 | 64.684 | 538 | 777 | 0 | 99 |
| WP_001572373.1 | Klebsiella_pneumoniae_SBY84561.1 | 64.684 | 538 | 777 | 0 | 99 |
| WP_001572373.1 | Klebsiella_pneumoniae_WP_065801616.1 | 64.684 | 538 | 777 | 0 | 99 |
| WP_001572373.1 | Escherichia_coli_ASF81896.1 | 64.684 | 538 | 777 | 0 | 99 |
| WP_001572373.1 | Enterobacteriaceae_WP_039026394.1 | 64.684 | 538 | 777 | 0 | 99 |
| WP_001572373.1 | Enterobacteriaceae_WP_087879616.1 | 64.684 | 538 | 776 | 0 | 99 |
| WP_001572373.1 | Shigella_sonnei_WP_094321595.1 | 64.684 | 538 | 776 | 0 | 99 |
| WP_001572373.1 | Aeromonas_popoffii_WP_042039220.1 | 65.985 | 538 | 776 | 0 | 99 |
| WP_001572373.1 | Aeromonas_salmonicida_WP_005321527.1 | 63.011 | 538 | 766 | 0 | 99 |
| WP_001572373.1 | Aeromonas_enteropelogenes_WP_042072031.1 | 61.673 | 514 | 715 | 0 | 95 |
| WP_001572373.1 | Aeromonas_sp._L_1B5_3_WP_043851325.1 | 58.755 | 514 | 698 | 0 | 95 |
| WP_001572373.1 | Aeromonas_sp._SCS5_WP_071911762.1 | 58.755 | 514 | 697 | 0 | 95 |
| WP_001572373.1 | Aeromonas_finlandiensis_WP_033138917.1 | 58.058 | 515 | 669 | 0 | 96 |
| WP_001572373.1 | Salmonella_sp._ASR73329.1 | 43.396 | 530 | 486 | 2.49E-170 | 98 |
| WP_001572373.1 | Stenotrophomonas_maltophilia_WP_017354816.1 | 43.831 | 543 | 479 | 9.34E-168 | 99 |
| WP_001572373.1 | Kosakonia_pseudosacchari_WP_097399671.1 | 43.542 | 542 | 478 | 4.51E-167 | 99 |
| WP_001572373.1 | Stenotrophomonas_sp._LMG_10879_WP_099818484.1 | 44.382 | 534 | 469 | 8.38E-164 | 98 |
| WP_001572373.1 | Rubrivivax_gelatinosus_IL144_BAL95649.1 | 44.8 | 500 | 467 | 8.21E-163 | 91 |
| WP_001572373.1 | Xanthomonas_retroflexus_WP_088099975.1 | 44.068 | 531 | 465 | 2.20E-162 | 98 |
| WP_001572373.1 | Stenotrophomonas_maltophilia_5BA-I-2_EVT72525.1 | 44.09 | 533 | 465 | 3.83E-162 | 98 |
| WP_001572373.1 | Stenotrophomonas_maltophilia_WP_100465422.1 | 44.007 | 534 | 464 | 1.11E-161 | 98 |
| WP_001572373.1 | Lampropedia_hyalina_WP_073356508.1 | 44.289 | 499 | 463 | 2.16E-161 | 91 |
| WP_001572373.1 | Stenotrophomonas_sp._RIT309_WP_051584849.1 | 43.503 | 531 | 462 | 5.47E-161 | 98 |
| WP_001572373.1 | Pectobacterium_carotovorum_WP_015840357.1 | 42.357 | 543 | 461 | 1.85E-160 | 99 |
| WP_001572373.1 | Stenotrophomonas_rhizophila_AHY58671.1 | 42.936 | 545 | 460 | 5.84E-160 | 99 |
| WP_001572373.1 | Kosakonia_sacchari_WP_065368351.1 | 43.853 | 545 | 456 | 1.42E-158 | 99 |
| WP_001572373.1 | Stenotrophomonas_maltophilia_WP_065179212.1 | 43.843 | 536 | 455 | 1.71E-157 | 98 |
| WP_001572373.1 | Klebsiella_WP_072310976.1 | 44.779 | 498 | 451 | 2.01E-156 | 91 |
| WP_001572373.1 | Atlantibacter_hermannii_WP_043865414.1 | 41.328 | 542 | 449 | 1.75E-155 | 99 |
| WP_001572373.1 | Xanthomonas_citri_pv._mangiferaeindicae_ASR42364.1 | 42.173 | 543 | 442 | 3.09E-153 | 99 |
| WP_001572373.1 | Stenotrophomonas_maltophilia_KUP00845.1 | 42.857 | 539 | 431 | 8.13E-148 | 99 |
| WP_001572373.1 | Stenotrophomonas_maltophilia_WP_099589211.1 | 43.043 | 539 | 428 | 5.12E-147 | 99 |
| WP_001572373.1 | Stenotrophomonas_maltophilia_WP_057500426.1 | 42.857 | 539 | 426 | 3.55E-146 | 99 |
| WP_001572373.1 | Gallibacterium_anatis_WP_013745741.1 | 40 | 535 | 423 | 8.23E-146 | 98 |
| WP_001572373.1 | Stenotrophomonas_maltophilia_KLN99845.1 | 46.61 | 472 | 425 | 1.26E-145 | 87 |
| WP_001572373.1 | Haemophilus_massiliensis_WP_032109569.1 | 37.594 | 532 | 412 | 2.03E-141 | 98 |
| WP_001572373.1 | Kingella_kingae_WP_003788372.1 | 37.893 | 541 | 405 | 6.87E-139 | 99 |
| WP_001572373.1 | Avibacterium_paragallinarum_WP_046097229.1 | 38.561 | 542 | 401 | 3.36E-137 | 99 |
| WP_001572373.1 | Neisseria_meningitidis_AKM91408.1 | 37.828 | 534 | 399 | 3.99E-136 | 98 |
| WP_001572373.1 | Mannheimia_WP_011199569.1 | 37.158 | 549 | 398 | 5.54E-136 | 99 |
| WP_001572373.1 | Necropsobacter_rosorum_WP_032093643.1 | 36.792 | 530 | 394 | 2.69E-134 | 98 |
| WP_001572373.1 | Salmonella_enterica_subsp._enterica_serovar_Paratyphi_B_ASK40562.1 | 33.213 | 554 | 337 | 3.81E-112 | 99 |
| WP_001572373.1 | Gammaproteobacteria_WP_049589868.1 | 36.262 | 535 | 325 | 1.12E-107 | 97 |
| WP_001572373.1 | Moraxella_pluranimalium_WP_078254299.1 | 34.27 | 534 | 312 | 8.22E-103 | 96 |
| WP_001572373.1 | Escherichia_coli_SBV31106.1 | 33.895 | 534 | 310 | 6.84E-102 | 96 |
| WP_001572373.1 | Escherichia_coli_WP_065419574.1 | 33.895 | 534 | 310 | 6.84E-102 | 96 |
| WP_001572373.1 | Psychrobacter_piscatorii_WP_058025526.1 | 33.65 | 526 | 309 | 1.63E-101 | 96 |
| WP_001572373.1 | Campylobacter_jejuni_CKG62629.1 | 36.842 | 513 | 306 | 1.71E-100 | 95 |
| WP_001572373.1 | Moraxella_sp._MSG47-C17_ASK49942.1 | 33.714 | 525 | 296 | 1.97E-96 | 94 |
| WP_001572373.1 | Paenibacillus_sophorae_WP_036596266.1 | 33.069 | 505 | 292 | 1.24E-94 | 91 |
| WP_001572373.1 | Enhydrobacter_aerosaccus_WP_007116571.1 | 32.871 | 505 | 291 | 4.18E-94 | 91 |
| WP_001572373.1 | Dichelobacter_nodosus_KNZ39428.1 | 32.649 | 536 | 274 | 5.41E-88 | 97 |
| WP_001572373.1 | Moraxella_catarrhalis_WP_003672704.1 | 33.333 | 480 | 258 | 1.11E-81 | 85 |

^a^Refers to papers describing *mcr*-3 (Yin, et al., MBio 8:e00543-17, 2017, doi: 10.1128/mBio.00543-17), *mcr*-4 (Carattoli, et al., Euro Surveill 22:30589, 2017, doi: 10.2807/1560-7917.ES.2017.22.31.30589), *mcr*-7 (Yang, et al., J Antimicrob Chemother 73:1791-1795, 2018, doi: https://doi.org/10.1093/jac/dky111), and *mcr*-8 (Wang, et al., Emerg Microbes Infect 7:122, 2018, doi: 10.1038/s41426-018-0124-z)

^b^Amino acid sequences of putative phosphoethanolamine transferases used in other papers describing novel *mcr* genes (n = 61) were downloaded using their NCBI Protein Accession numbers and used as database to which *mcr*-9 was aligned using the command-line implementation of protein blast (blastp) version 2.7.1 and default alignment and scoring parameters (C. Camacho, et al., BMC Bioinformatics 10:421, 2009, doi: 10.1186/1471-2105-10-421).

^c^Refers to *mcr-*9 (NCBI Protein Accession WP_001572373.1), which was treated as the query for blastp.

^d^Refers to the identifier used in this study (taxonomic information, followed by NCBI Protein Accession number)
