# Supplementary material for: Identification of Novel Mobilized Colistin Resistance Gene mcr-9 in a Multidrug-Resistant, Colistin-Susceptible Salmonella enterica Serotype Typhimurium Isolate
Source: mBio. 2019 May 7;10(3):e00853-19. doi: 10.1128/mBio.00853-19 (PMC6509194; doi:10.1128/mBio.00853-19)
Supplement: FIG S1 [file mBio.00853-19-sf001.pdf]

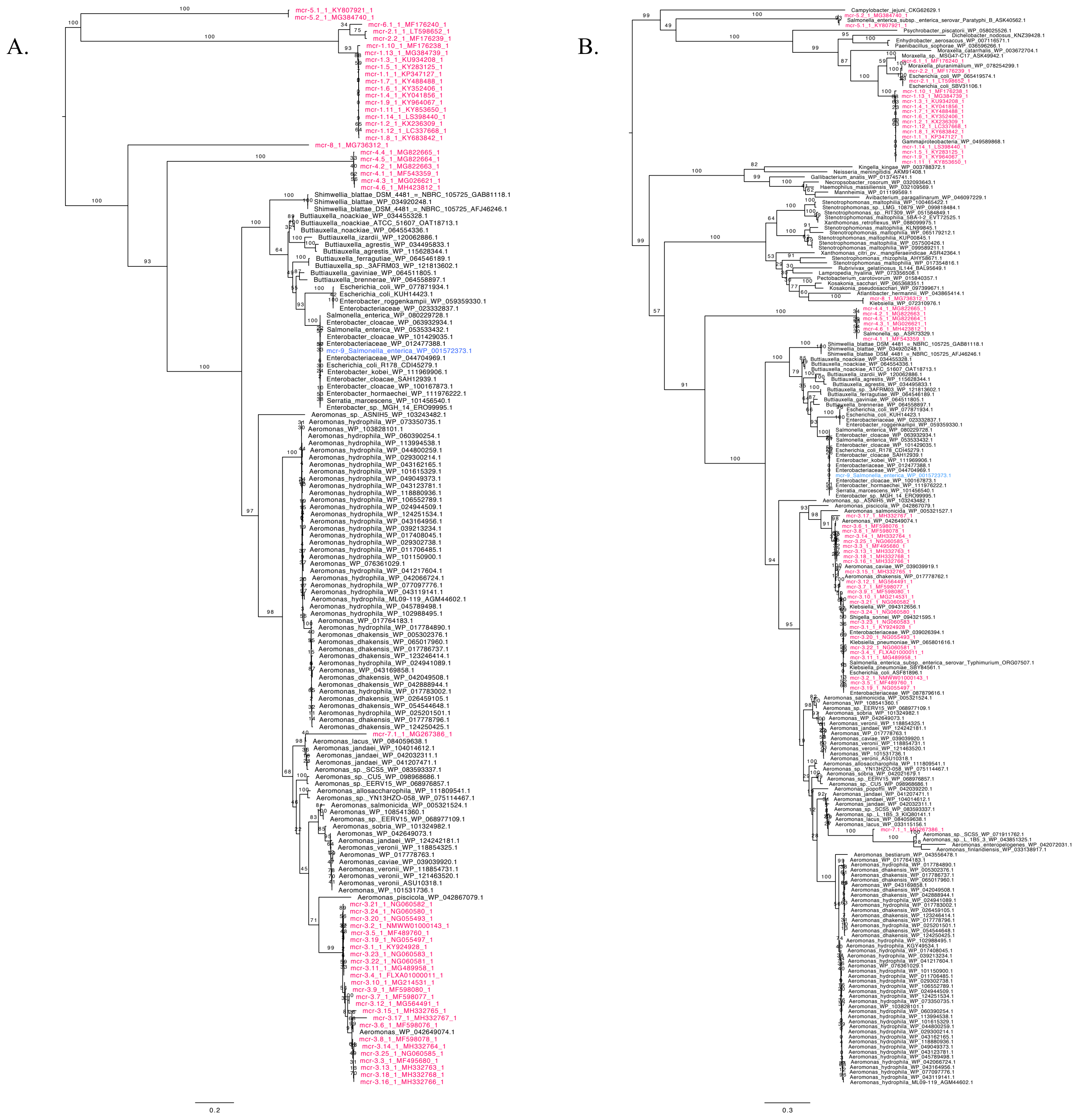

Supplemental Figure S1. Maximum likelihood phylogenies constructed using the amino acid sequences of novel mobilized colistin resistance gene *mcr*-9 (in blue), plus all (n = 52) previously described *mcr* genes (*mcr*-1, -2, -3, -4, -5, -6, -7, and -8) available in ResFinder (accessed January 22, 2019; in pink), as well as (A) the 100 top hits produced when *mcr*-9 was queried against NCBI's non-redundant protein sequence (nr) database using the protein blast (blastp) webserver (accessed January 22, 2019) and default parameters (152 total sequences; *mcr*-9's self-match was excluded, as it was already present in the nr database), as well as (B) amino acid sequences of 61 putative phosphoethanolamine transferases used in other papers describing novel *mcr* genes (213 total sequences). RAxML version 8.2.12 was used to construct the phylogenies, which were annotated with FigTree version 1.4.3. The phylogenies are rooted at the midpoint, with branch lengths reported in substitutions per site. Branch labels correspond to bootstrap support percentages out of 1,000 replicates.
