# Supplementary material for: Identification of Novel Mobilized Colistin Resistance Gene mcr-9 in a Multidrug-Resistant, Colistin-Susceptible Salmonella enterica Serotype Typhimurium Isolate
Source: mBio. 2019 May 7;10(3):e00853-19. doi: 10.1128/mBio.00853-19 (PMC6509194; doi:10.1128/mBio.00853-19)
Supplement: TABLE S5 [file mBio.00853-19-st005.docx]

**Supplemental Table S5.** Minimum inhibitory concentration (MIC) profiles of colistin against *Salmonella* strains tested in this study.

| **Study ID** | **FMT ID^a^** | **NCBI RefSeq Accession^b^** | **Taxonomy** | ***mcr* Gene(s)^c^** | ***pmrA*^d^** | ***pmrB*^e^** | **MIC (mg/L)^f^** |
| --- | --- | --- | --- | --- | --- | --- | --- |
| HUM_TYPH_NY_09_R8_3574 | FSL R8-3574 | NA | *Salmonella enterica* serotype Typhimurium | None | WT | WT | 0.125 |
| HUM_TYPH_WA_08_R9_3269 | FSL R9-3269 | GCF_002091285.1 | *Salmonella enterica* serotype Typhimurium^g^ | *mcr*-3 | WT | WT | 4 |
| HUM_TYPH_WA_10_R9_3274 | FSL R9-3274 | GCF_002091095.1 | *Salmonella enterica* serotype Typhimurium | *mcr*-9 | WT | WT | 0.25 – 0.5 |

^a^Food Microbe Tracker (FMT) identification number (<http://www.foodmicrobetracker.com>; P. Vangay, E.B. Fugett, Q. Sun, and M. Wiedmann, J Food Prot 76(2):283-294, 2013, doi: 10.4315/0362-028X.JFP-12-276)

^b^NCBI RefSeq Accession number; denoted by NA if not currently publicly available

^c^Detected using translated nucleotide blast (tblastn; C. Camacho, et al., BMC Bioinformatics 10:421, 2009, doi: 10.1186/1471-2105-10-421) as implemented in BTyper version 2.3.2 (L.M. Carroll, J. Kovac, R.A. Miller, and M. Wiedmann, Appl Environ Microbiol 83(17): e01096-17, 2017, doi: 10.1128/AEM.01096-17) and the 52 *mcr* nucleotide sequences available in ResFinder (accessed January 22, 2019; ; E. Zankari, et al., J Antimicrob Chemother 67(11): 2640-2644, 2012, doi: 10.1093/jac/dks261), translated into amino acid sequences using EMBOSS Transeq (reading frame 1; https://www.ebi.ac.uk/Tools/st/emboss_transeq/)

^d^Nucleotide and amino acid substitutions in *pmrA* (*basR*); the wild-type (WT) *pmrA* of NCBI Reference Genome *S.* Typhimurium str. LT2 (NCBI Gene ID 1255818) was aligned to *pmrA* alleles detected in each assembly using nucleotide blast (blastn) to ensure that no potential colistin resistance-conferring mutations were present. Genes that matched with 100% identity and coverage (i.e., had no nucleotide or amino acid substitutions) are denoted by WT.

^e^Nucleotide and amino acid substitutions in *pmrB* (*basS*); the wild-type (WT) *pmrB* of NCBI Reference Genome *S.* Typhimurium str. LT2 (NCBI Gene ID 1255817) was aligned to *pmrB* alleles detected in each assembly using nucleotide blast (blastn) to ensure that no potential colistin resistance-conferring mutations were present. Genes that matched with 100% identity and coverage (i.e., had no nucleotide or amino acid substitutions) are denoted by WT.

^f^Minimum inhibitory concentration (MIC) determined using the broth microdilution method described by the European Committee on Antimicrobial Susceptibility Testing (EUCAST; <http://www.eucast.org>)

^g^Reported as Typhimurium using traditional serotyping and antigen-based prediction in SISTR version 1.0.2 (C.E. Yoshida, et al., PLoS One 11(1): e0147101, 2016, doi: doi: 10.1371/journal.pone.0147101); reported as 4,5,12:i:- by core genome multi-locus sequence typing (cgMLST)-based prediction in SISTR
