# Supplementary material for: Identification of Novel Mobilized Colistin Resistance Gene mcr-9 in a Multidrug-Resistant, Colistin-Susceptible Salmonella enterica Serotype Typhimurium Isolate
Source: mBio. 2019 May 7;10(3):e00853-19. doi: 10.1128/mBio.00853-19 (PMC6509194; doi:10.1128/mBio.00853-19)
Supplement: TEXT S1 [file mBio.00853-19-s0001.docx]

**Supplemental Text**

Detailed descriptions of experimental methods.

**Cloning of *mcr* genes.** The coding region of *mcr*-3 was amplified from strain FSL R9-3269 using primers GBS55-1 (ATAggatcctaaggaggctTCATAATGAGTGATGTCTCGTT) and GBS55-2 (ATActgcagGAAATACACGGACAATAGTAATA). The coding region of *mcr*-9 was amplified from strain FSL R9-3274 using primers GBS55-5 (ATAggatcctaaggaggctcatcaatgtttttactggtttac) and GBS55-4 (atactgcagCGTAAGGCTTTTTGTTATACTAA). A canonical ribosome-binding site (underlined) was incorporated in the forward primer 7bp upstream of the start codon (bold underlined) to optimize expression (R.L. Vellanoweth and J.C. Rabinowitz, Mol Microbiol 6(9):1105-1114, 1992, doi: https://doi.org/10.1111/j.1365-2958.1992.tb01548.x). PCR products were cloned using *BamH*I and *Pst*I restriction enzymes (bold) in the pLIV2 vector (D.E. Higgins, C. Buchrieser, and N.E. Freitag, p. 620-633, *in* V. Fischetti, R. Novick, J. Ferretti, D. Portnoy, J. Rood, ed., *Gram-Positive Pathogens*, 2nd ed., 2006, doi: 10.1128/9781555816513.ch51). Expression of the *mcr* genes was derived from the IPTG-induced pSPAC/lacOid promoter in the pLIV2 vector in *E. coli* NEB5α. No plasmid extraction methods were performed.

**Colistin killing assay.** *E. coli* harboring the pLIV2 empty vector (negative control), pLIV2::*mcr*-3 (positive control), or pLIV2::*mcr*-9 were used in colistin killing assays. Tubes containing 5 ml of Mueller Hinton II Broth media (MH-II, BD cat number 212322) supplemented with IPTG were inoculated from overnight cultures (12-14h) and incubated at 37ºC with vigorous shaking to OD_600_ of 0.4-0.6. Killing assays were carried out as previously described (H. Lee, F.F. Hsu, J. Turk, and E.A. Groisman, J Bacteriol 186(13):4124-4133, 2004, doi: 10.1128/JB.186.13.4124-4133.2004). Briefly, colistin was added at concentrations of 0, 1, 2, 2.5, or 5 mg/L, and the bacteria were incubated at 37⁰C for 1 h. Samples were diluted in PBS and plated on LB agar plates for the determination of CFU. Log CFU reduction after treatment was calculated from three independent biological replicates. While *E. coli* harboring pLIV2::*mcr*-3 grew to the same levels as the pLIV2 containing strain, the pLIV2::*mcr*-9 construct caused growth inhibition of *E. coli* in the presence of 1mM IPTG. To reduce the possible toxicity that resulted from *mcr*-9 over-expression, IPTG concentrations were reduced to 0.4mM for all strains at colistin concentrations of 1 and 2 mg/L.

***In silico* structural modeling.** Structural modeling of different proteins encoded by representatives of the *mcr* family and sub-family was done using the Phyre2 server (L.A. Kelley, S. Mezulis, C.M. Yates, M.N. Wass, and M.J. Sternberg, Nat Protoc 10(6):845-858, 2015, doi: 10.1038/nprot.2015.053) based on lipooligosaccharide phosphoethanolamine transferase, EptA (A. Anandan, G.L. Evans, K. Condic-Jurkic, M.L. O’Mara, C.M. John, N.J. Phillips, G.A. Jarvis, S.S. Wills, K.A. Stubbs, I. Moraes, C.M., Kahler, and A. Vrielink, Proc Natl Acad Sci USA 114(9):2218-2223, 2017, doi: 10.1073/pnas.1612927114). The structures were viewed and annotated using UCSF Chimera (E.F. Pettersen, T.D. Goddard, C.C. Huang, G.S. Couch, D.M. Greenblatt, E.C. Meng, and T.E. Ferrin, J Comput Chem 25(13):1605-1612, 2004, doi: https://doi.org/10.1002/jcc.20084). The ESPript 3 server was used to show sequence similarity and secondary structure information (X. Robert and P. Gouet, Nucleic Acids Res 42:W320-W324, 2014, doi: 10.1093/nar/gku316). Structural relationships between different Mcr proteins were performed using the Dali server (L. Holm and L.M. Laakso, Nucleic Acids Res 44:W351-W355, 2016, doi: 10.1093/nar/gkw357). Structural similarity matrices and cluster dendrograms are based on the *Z* score comparisons calculated from a Dali all-against-all analysis (L. Holm and L.M. Laakso, Nucleic Acids Res 44:W351-W355, 2016, doi: 10.1093/nar/gkw357).

**Promoter analysis.** Promoter elements and conserved DNA motifs were queried in the upstream sequences of 321 *mcr*-9 coding regions (Supplemental Table S6). Contigs listed in Supplemental Table S6 were downloaded from NCBI’s Nucleotide database using the Batch Entrez web server (<https://www.ncbi.nlm.nih.gov/sites/batchentrez>). Twenty-two of the contigs on which *mcr*-9 was detected were not included in the promoter analysis, as they could not be accessed in NCBI’s Nucleotide database via the Batch Entrez web server (see “Contigs excluded from promoter analysis” section below). The -35 and -10 promoter elements were predicted using PromoterHunter (L. Klucar, M. Stano, and M. Hajduk, Nucleic Acids Res 38(Database issue): D366–D370, 2010, doi: 10.1093/nar/gkp911) and matrices for -35 and -10 of *E. coli* σ^70^ recognition sequences. Conserved motifs in different *mcr*-9 promoter regions were identified using the DMINDA web server (<http://bmbl.sdstate.edu/DMINDA2/annotate.php>) (J. Yang, X. Chen, McDermaid A, and Q. Ma, Bioinformatics 33(16): 2586-2588, 2017, doi: 10.1093/bioinformatics/btx223).

**Contigs excluded from promoter analysis.** The following accession numbers correspond to contigs on which *mcr*-9 was detected that were excluded from the promoter analysis (see “Promoter analysis” section above):

QFQQ01000156.1

MKVD01000078.1

RTSZ01000082.1

UWVU01000002.1

UWWX01000002.1

UWXQ01000002.1

LYPV01000097.1

RLTW01000072.1

RLTW01000096.1

RMYC01000125.1

RMYC01000099.1

ROVE01000055.1

ROVE01000082.1

RSQS01000061.1

RUFV01000118.1

RUCL01000124.1

RUCL01000155.1

RSGR01000007.1

RSHP01000061.1

RSHP01000106.1

PEHB01000007.1

DOZY01000196.1.
