# Supplementary material for: Identification of Novel Mobilized Colistin Resistance Gene mcr-9 in a Multidrug-Resistant, Colistin-Susceptible Salmonella enterica Serotype Typhimurium Isolate
Source: mBio. 2019 May 7;10(3):e00853-19. doi: 10.1128/mBio.00853-19 (PMC6509194; doi:10.1128/mBio.00853-19)
Supplement: FIG S2 [file mBio.00853-19-sf002.pdf]

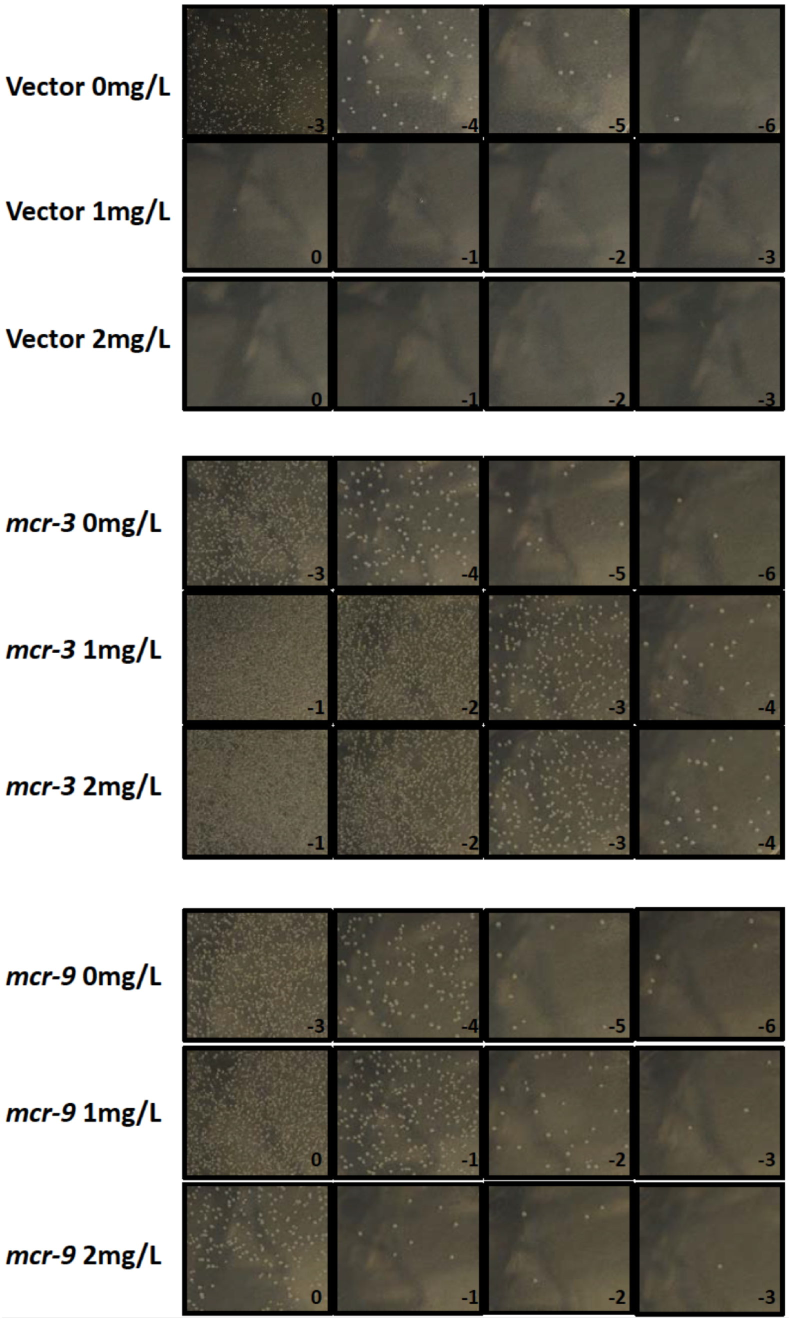

Supplemental Figure S2. Selected images associated with colistin killing assay of *E. coli* NEB5α harboring a pLIV2 empty vector (negative control), *mcr*-3 (positive control), or *mcr*-9, expressed under the control of the IPTG controlled SPAC/lacOid promoter. Cells were grown in MH-II media with IPTG to mid-exponential phase. Colistin was added at concentrations of 0, 1, 2, 2.5, or 5 mg/L, and the bacteria were incubated at 37°C for 1 h. The samples were diluted in PBS and plated on LB agar plates for the determination of CFU.
