# Supplementary material for: Identification of Novel Mobilized Colistin Resistance Gene mcr-9 in a Multidrug-Resistant, Colistin-Susceptible Salmonella enterica Serotype Typhimurium Isolate
Source: mBio. 2019 May 7;10(3):e00853-19. doi: 10.1128/mBio.00853-19 (PMC6509194; doi:10.1128/mBio.00853-19)
Supplement: TABLE S6 [file mBio.00853-19-st006.docx]

**Supplemental Table S6.** Location of *mcr*-9 on contigs for 335 genome assemblies.

| **Genome (Assembly Level)^a^** | **BioSample Accession** | **Isolation Source^b^** | **Country of Isolation^c^** | **Year of Isolation^d^** | **Contig of *mcr*-9^e^** | **Additional Plasmid Replicons and AMR Genes Detected on Contig^f^** |
| --- | --- | --- | --- | --- | --- | --- |
| Citrobacter_farmeri_GCF_002249995.1_ASM224999v1_genomic (Complete Genome) | SAMN07452765 | Human (rectal) | Australia (Victoria) | 2015 | NZ_CP022696.1 (Plasmid pAUSMDU8141-1) | IncHI2A_1,IncHI2_1,TrfA_1,*aadA2_1,sul1_5,qnrA1_1,sul1_5,qnrA1_1,sul1_5,dfrA18_1,strA_1,aph(6)-Id_1,tet(D)_1,blaTEM-1B_1,mph(A)_2,mph(A)_1,aac(3)-IId_1,blaTEM-1B_1,sul1_5,ARR-3_4,catB3_1,blaOXA-1_1,aac(6')Ib-cr_1,blaSHV-12_1,catA2_1* |
| Citrobacter_freundii_GCA_003243445.1_ASM324344v1_genomic (Contig)* | SAMN07426513 | Environmental (hospital) | USA (Pennsylvania: Pittsburgh) | 2013 | QFQQ01000156.1 | NONE |
| Citrobacter_freundii_GCF_000937455.2_ASM93745v2_genomic (Scaffold) | SAMN03316839 | Human | USA | 2012 | NZ_JYFY02000009.1 | *strA_1,aph(6)-Id_1* |
| Citrobacter_freundii_GCF_001482545.1_ASM148254v1_genomic (Scaffold) | SAMN04124434 | Human (pleural fluid) | Denmark | 2014 | NZ_LLJO01000053.1 | NONE |
| Citrobacter_freundii_GCF_001482575.1_ASM148257v1_genomic (Scaffold) | SAMN04124438 | Human (urine) | Denmark | 2015 | NZ_LLJR01000067.1 | NONE |
| Citrobacter_freundii_GCF_004024405.1_ASM402440v1_genomic (Contig) | SAMN09289737 | Environmental (water) | USA | 2017 | NZ_QKPL01000104.1 | NONE |
| Cronobacter_malonaticus_GCF_002093915.1_ASM209391v1_genomic (Scaffold) | SAMN03782455 | Human (cerebrospinal fluid) | China (Hubei) | 2014 | NZ_LGRM01000052.1 | NONE |
| Cronobacter_malonaticus_GCF_002978485.1_ASM297848v1_genomic (Scaffold) | SAMN07483918 | Food | China | 2007 | NZ_NRNJ01000011.1 | NONE |
| Cronobacter_sakazakii_GCF_001309235.1_ASM130923v1_genomic (Scaffold) | SAMN02840818 | Food (infant formula) | France | 1994 | NZ_JOLS01000063.1 | *fos_2* |
| Cronobacter_sakazakii_GCF_002094675.1_ASM209467v1_genomic (Contig) | SAMN05413002 | Food (deli meat) | China (Jiangxi: Nanchang) | 2012 | NZ_MBRZ01000024.1 | NONE |
| Cronobacter_sakazakii_GCF_002107695.1_ASM210769v1_genomic (Contig) | SAMN04329593 | Human | Ireland | NA | NZ_NCWF01000071.1 | NONE |
| Cronobacter_sakazakii_GCF_002942245.1_ASM294224v1_genomic (Contig) | SAMN08397412 | Food (walnuts) | USA (Maryland) | 2015 | NZ_PTOM01000004.1 | *fos_2* |
| Cronobacter_sakazakii_GCF_002974915.1_ASM297491v1_genomic (Contig) | SAMN08611280 | Food (whey protein concentrate) | USA (Illinois) | 2004 | NZ_PVDA01000012.1 | NONE |
| Cronobacter_sakazakii_GCF_002974975.1_ASM297497v1_genomic (Contig) | SAMN08611386 | Food (whey protein concentrate) | USA (Illinois) | 2004 | NZ_PVDH01000018.1 | NONE |
| Cronobacter_sakazakii_GCF_002977765.1_ASM297776v1_genomic (Scaffold) | SAMN07483960 | Food | China | 2007 | NZ_NRLT01000074.1 | NONE |
| Cronobacter_sakazakii_GCF_002977865.1_ASM297786v1_genomic (Scaffold) | SAMN07483953 | Food | China | 2013 | NZ_NRMA01000210.1 | NONE |
| Cronobacter_sakazakii_GCF_003207135.1_ASM320713v1_genomic (Contig) | SAMN09273080 | Human | Canada | 2006 | NZ_QISG01000021.1 | NONE |
| Cronobacter_sakazakii_GCF_003955925.1_ASM395592v1_genomic (Complete Genome) | SAMN08928172 | Human (brain abscess fluid) | China | 2015 | NZ_CP028975.1 (Plasmid pGW1) | IncHI2A_1,TrfA_1,IncHI2_1,*qnrB4_1,blaDHA-1_1,sul1_5,blaSFO-1_1,mph(A)_2,mph(A)_1,sul1_5,aadA2_1,dfrA12_1,aac(3)-IId_1,blaTEM-1B_1,dfrA18_1,strA_1,aph(6)-Id_1,aph(3')-Ia_10,tet(D)_1,catA2_1,aac(6')-IIc_1,aac(3)-IIb_1,ere(A)_2,sul1_5* |
| Enterobacter_asburiae_GCF_000534055.1_Ente_cloa_UCI_49_V1_genomic (Scaffold) | SAMN02356629 | Human (urine) | USA (California: Irvine) | NA | NZ_KI973092.1 | *blaACT-6_1,fosA_7,oqxA_1,oqxB_1* |
| Enterobacter_asburiae_GCF_000965825.1_ASM96582v1_genomic (Contig)^g^ | SAMN03277192 | Human | Colombia | 2009 | NZ_JZKP01000092.1 | NONE |
| Enterobacter_asburiae_GCF_000966005.1_ASM96600v1_genomic (Contig) | SAMN03277191 | Human | Colombia | 2009 | NZ_JZKO01000109.1 | NONE |
| Enterobacter_asburiae_GCF_001053615.1_ASM105361v1_genomic (Contig) | SAMN03196991 | Human | USA (Washington) | NA | NZ_JWFR01000124.1 | NONE |
| Enterobacter_asburiae_GCF_001057935.1_ASM105793v1_genomic (Contig) | SAMN03197886 | Human | USA (Washington) | NA | NZ_JUXG01000136.1 | NONE |
| Enterobacter_asburiae_GCF_001060045.1_ASM106004v1_genomic (Scaffold) | SAMN03198217 | Human | USA (Washington) | NA | NZ_JUKN01000151.1 | NONE |
| Enterobacter_asburiae_GCF_001482465.1_ASM148246v1_genomic (Contig) | SAMN04124432 | Human (urine) | Denmark | 2013 | NZ_LLJM01000059.1 | NONE |
| Enterobacter_asburiae_GCF_001482565.1_ASM148256v1_genomic (Scaffold) | SAMN04124437 | Human (sputum) | NA | 2014 | NZ_LLJQ01000072.1 | NONE |
| Enterobacter_asburiae_GCF_001482625.1_ASM148262v1_genomic (Scaffold) | SAMN04124433 | Human (urine) | Denmark | 2014 | NZ_LLJN01000058.1 | NONE |
| Enterobacter_asburiae_GCF_001525055.1_ASM152505v1_genomic (Contig) | SAMN04430996 | Human (urine) | Colombia | 2014 | NZ_LRJQ01000059.1 | NONE |
| Enterobacter_asburiae_GCF_002333885.1_ASM233388v1_genomic (Scaffold) | SAMD00089475 | Human | Japan (Kantō: Tokyo) | 2010 | NZ_BEEV01000081.1 | NONE |
| Enterobacter_asburiae_GCF_002334005.1_ASM233400v1_genomic (Scaffold) | SAMD00089481 | Human | Japan (Kantō: Tokyo) | 2010 | NZ_BEFB01000108.1 | NONE |
| Enterobacter_asburiae_GCF_003964705.1_ASM396470v1_genomic (Contig) | SAMN08932714 | Human | China (Sichuan: Chengdu) | 2017 | NZ_RXPU01000066.1 | NONE |
| Enterobacter_asburiae_GCF_004024095.1_ASM402409v1_genomic (Scaffold) | SAMN09289753 | Environmental (water) | USA | 2017 | NZ_QKOV01000095.1 | NONE |
| Enterobacter_cloacae_complex_sp._ECNIH12_GCF_002919685.1_ASM291968v1_genomic (Contig) | SAMN06040404 | Environmental (wastewater and/or sludge) | USA (Maryland: Bethesda) | 2016 | NZ_PQKV01000099.1 | NONE |
| Enterobacter_cloacae_complex_sp._ECNIH14_GCF_002918755.1_ASM291875v1_genomic (Contig) | SAMN06040414 | Environmental (wastewater and/or sludge) | USA (Maryland: Bethesda) | 2016 | NZ_PQKZ01000055.1 | NONE |
| Enterobacter_cloacae_complex_sp._ECNIH8_GCF_002918735.1_ASM291873v1_genomic (Contig) | SAMN04287063 | Human | USA (Maryland: Bethesda) | 2011 | NZ_PQKY01000046.1 | NONE |
| Enterobacter_cloacae_complex_sp._GCF_003225955.1_ASM322595v1_genomic (Contig) | SAMN09104662 | Human (urine) | Spain (Barcelona: Barcelona) | 2017 | NZ_QKNF01000001.1 | *fosA_1,blaACT-9_1,aac(2')-IIa_1* |
| Enterobacter_cloacae_complex_sp._GCF_900322725.1_C45_genomic (Complete Genome) | SAMEA104700141 | Human | France | 2014 | NZ_LT991958.1 (Plasmid pC45-VIM4) | IncHI2A_1,IncHI2_1,TrfA_1,*blaVIM-4_1,aac(6')-Il_1,dfrA1_30,aadA1_5,sul1_5,blaTEM-1B_1,ant(2'')-Ia_1,aadA2_1,sul1_5,qnrA1_1,sul1_5,tet(A)_6* |
| Enterobacter_cloacae_complex_sp._TREC1_GCF_002858545.1_ASM285854v1_genomic (Scaffold) | SAMN08156302 | Human (blood) | NA | 2017 | NZ_PJZE01000054.1 | NONE |
| Enterobacter_cloacae_ECNIH2_GCF_000724505.1_ASM72450v1_genomic (Complete Genome) | SAMN02713682 | Environmental (sink drain) | USA | 2012 | NZ_CP008825.1 (Plasmid pKPC-272) | IncHI2A_1,IncHI2_1,TrfA_1,*blaTEM-1B_1,blaKPC-3_1,ant(2'')-Ia_1,catB3_1,sul1_5,aph(3')-Ia_10* |
| Enterobacter_cloacae_GCA_002740835.1_ASM274083v1_genomic (Scaffold)* | SAMN07452577 | Human (blood) | Romania (Mureș: Târgu Mureș) | 2013 | CM008909.1 (Plasmid p20ES-288) | IncHI2A_1,TrfA_1,IncHI2_1,*blaSHV-12_1,aadA2_1,sul1_5,qnrA1_1,sul1_5,dfrA18_1,strA_1,aph(6)-Id_1,catA2_1,blaTEM-1B_1* |
| Enterobacter_cloacae_GCF_002237465.1_ASM223746v1_genomic (Complete Genome) | SAMN06765483 | Human (endotracheal tube) | Australia (Queensland: Brisbane) | 2015 | NZ_CP022533.1 (Plasmid pMS7884A) | IncHI2_1,TrfA_1,IncHI2A_1,*blaTEM-1B_1,tet(D)_1,catA2_1,aph(6)-Id_1,strA_1,dfrA18_1,sul1_5,catB3_1,blaIMP-4_1,blaTEM-1B_1,aac(3)-IId_1,mph(A)_2,mph(A)_1,sul1_5,qnrB2_1,sul1_5,ARR-3_4,catB3_1,blaOXA-1_1,aac(6')Ib-cr_1* |
| Enterobacter_cloacae_GCF_003046225.1_ASM304622v1_genomic (Contig) | SAMN08848070 | Environmental (wastewater) | Brazil (Amazonas: Manaus) | 2016 | NZ_PZPP01000030.1 | NONE |
| Enterobacter_cloacae_GCF_003176615.1_ASM317661v1_genomic (Contig) | SAMD00115713 | Human | NA | 2010 | NZ_BGMH01000064.1 | NONE |
| Enterobacter_cloacae_GCF_003204095.1_ASM320409v1_genomic (Complete Genome) | SAMN04014995 | NA | NA | NA | NZ_CP029717.1 (Plasmid unnamed4) | IncHI2_1,TrfA_1,IncHI2A_1,*aac(6')-IIc_1,aac(3)-IIb_1,ere(A)_2,sul1_5,sul2_2,blaSHV-12_1,catA2_1,blaTEM-1B_1,catA2_1,tet(D)_1,aadA2_1,sul1_5,qnrB2_1,sul1_5,dfrA18_1,strA_1,aph(6)-Id_1,aadA2_1,sul1_5,qnrB2_1,sul1_5,dfrA18_1,strA_1,aph(6)-Id_1* |
| Enterobacter_cloacae_GCF_003264955.1_ASM326495v1_genomic (Complete Genome) | SAMN09425565 | Human (sputum) | China (Shandong) | 2011 | NZ_CP030080.1 (Plasmid pIMP-20710) | IncHI2A_1,TrfA_1,IncHI2_1,*dfrA12_1,aadA2_1,sul1_5,floR_1,tet(G)_2,blaIMP-34_1* |
| Enterobacter_cloacae_GCF_003289825.1_ASM328982v1_genomic (Scaffold) | SAMN09435809 | Human (rectal) | France | 2016 | NZ_QMDH01000098.1 | NONE |
| Enterobacter_cloacae_GCF_900447335.1_32407_F01_genomic (Contig) | SAMEA2709018 | NA | NA | 2017 | NZ_UGIN01000001.1 | *blaACT-14_1,fosA_1,oqxB_1,oqxA_1* |
| Enterobacter_cloacae_subsp._cloacae_GCF_001022695.1_ASM102269v1_genomic (Contig) | SAMN03732719 | Human | USA (North Carolina: Durham) | 2007 | NZ_LEDL01000039.1 | NONE |
| Enterobacter_cloacae_subsp._cloacae_GCF_001525175.1_ASM152517v1_genomic (Contig) | SAMN04431024 | Human | Vietnam | 2014 | NZ_LRIO01000027.1 | NONE |
| Enterobacter_hormaechei_GCA_002740875.1_ASM274087v1_genomic (Contig) | SAMN07452566 | Human (wound) | Romania (Mureș: Târgu Mureș) | 2013 | CM008898.1 (Plasmid p22ES-287) | IncHI2_1,TrfA_1,IncHI2A_1,*tet(D)_1,blaSHV-12_1,qnrA1_1,sul1_5,dfrA18_1,strA_1,aph(6)-Id_1,blaTEM-1B_1,catA2_1* |
| Enterobacter_hormaechei_GCA_003408555.1_ASM340855v1_genomic (Complete Genome) | SAMN04448506 | Human (rectal) | NA | 2013 | CP031568.1 (Plasmid pSHV12-1301491) | *sul1_5,catA2_1,blaSHV-12_1,sul2_2,sul1_5,ere(A)_2,aac(3)-IIb_1,aac(6')-IIc_1,aph(6)-Id_1,strA_1,dfrA18_1,sul1_5,qnrB2_1,sul1_5,aadA2_1* |
| Enterobacter_hormaechei_GCA_003408575.1_ASM340857v1_genomic (Complete Genome) | SAMN04448498 | Human (rectal) | NA | 2015 | CP031571.1 (Chromosome) | *oqxA_1,oqxB_1,aph(6)-Id_1,strA_1,blaACT-7_1* |
| Enterobacter_hormaechei_GCF_000692235.1_Ente_cloa_BIDMC_66_V1_genomic (Scaffold) | SAMN02581287 | Human (tissue) | USA (Massachusetts: Boston) | 2013 | NZ_KK736221.1 | *tet(B)_2,blaKPC-4_1* |
| Enterobacter_hormaechei_GCF_000770745.1_ASM77074v1_genomic (Scaffold) | SAMN03013105 | Human (endotracheal aspirate) | Australia (Queensland: Brisbane) | 2013 | NZ_JRFQ01000065.1 | NONE |
| Enterobacter_hormaechei_GCF_000938355.2_ASM93835v2_genomic (Scaffold) | SAMN03316841 | Human | USA | 2013 | NZ_JYGA02000002.1 | IncHI2A_1,TrfA_1,IncHI2_1,*blaCTX-M-9_1,sul1_5,aadA2_1,dfrA16_1* |
| Enterobacter_hormaechei_GCF_000952475.1_ASM95247v1_genomic (Scaffold) | SAMN03333160 | Human (peritoneum) | USA (Minnesota: Granite Township) | 2011 | NZ_JYMJ01000105.1 | NONE |
| Enterobacter_hormaechei_GCF_002151825.1_ASM215182v1_genomic (Scaffold) | SAMN04521878 | Human (blood) | USA (Massachusetts: Boston) | 2015 | NZ_NGRJ01000002.1 | IncHI2A_1,TrfA_1,IncHI2_1,*sul1_5,aadA2_1,aph(3')-Ia_7,sul1_5,ant(3'')-Ia_1,dfrA8_1,catA1_1,aph(6)-Id_1,strA_1,dfrA18_1,sul1_5,qnrB2_1,sul1_5* |
| Enterobacter_hormaechei_GCF_002151915.1_ASM215191v1_genomic (Scaffold) | SAMN04521926 | Human | USA (Massachusetts: Boston) | 2015 | NZ_NGRO01000002.1 | IncHI2A_1,TrfA_1,IncHI2_1,*dfrA18_1,strA_1,aph(6)-Id_1,blaSHV-12_1,blaTEM-122_1,aadA2_1,qnrB2_1,sul1_5* |
| Enterobacter_hormaechei_GCF_002152945.1_ASM215294v1_genomic (Scaffold) | SAMN04521882 | Human | USA (Massachusetts: Boston) | 2015 | NZ_NGRK01000002.1 | IncHI2_1,TrfA_1,IncHI2A_1,*blaKPC-4_1* |
| Enterobacter_hormaechei_GCF_002208275.1_ASM220827v1_genomic (Contig) | SAMN07234405 | Human (blood) | Australia (South Australia: Adelaide) | 2017 | NZ_NJDA01000026.1 | NONE |
| Enterobacter_hormaechei_GCF_002264185.1_ASM226418v1_genomic (Contig) | SAMN07345016 | Human (blood) | China (Anhui) |  | NZ_NPGJ01000019.1 | IncFIB(pECLA)_1_pECLA,IncFII(pECLA)_1_pECLA |
| Enterobacter_hormaechei_GCF_002334225.1_ASM233422v1_genomic (Scaffold) | SAMD00089493 | Human | Japan (Kantō: Tokyo) | 2010 | NZ_BEFN01000079.1 | NONE |
| Enterobacter_hormaechei_GCF_002416795.1_ASM241679v1_genomic (Scaffold) | SAMN06106854 | Human | South Africa (Gauteng: Pretoria) | 2013 | NZ_NXJH01000131.1 | NONE |
| Enterobacter_hormaechei_GCF_002417315.1_ASM241731v1_genomic (Contig) | SAMN06106860 | Human (blood) | South Africa (Gauteng: Pretoria) | 2013 | NZ_NXJN01000053.1 | NONE |
| Enterobacter_hormaechei_GCF_002510085.1_ASM251008v1_genomic (Contig) | SAMN06106888 | Human (sputum) | South Africa (Gauteng: Pretoria) | 2013 | NZ_NXKO01000060.1 | NONE |
| Enterobacter_hormaechei_GCF_002785645.1_ASM278564v1_genomic (Scaffold) | SAMN06767791 | Human | China (Zhejiang: Hangzhou) | NA | NZ_NEEK01000044.1 | *blaCTX-M-9_1* |
| Enterobacter_hormaechei_GCF_002785655.1_ASM278565v1_genomic (Scaffold) | SAMN06767790 | Human | China (Zhejiang: Hangzhou) | NA | NZ_NEEL01000094.1 | NONE |
| Enterobacter_hormaechei_GCF_002785805.1_ASM278580v1_genomic (Scaffold) | SAMN06767780 | Human (sputum) | China (Zhejiang: Hangzhou) | 2011 | NZ_NEEV01000062.1 | NONE |
| Enterobacter_hormaechei_GCF_002795185.1_ASM279518v1_genomic (Contig) | SAMN07355852 | Human (blood) | China | 2017 | NZ_NMVR01000102.1 | NONE |
| Enterobacter_hormaechei_GCF_002878435.1_ASM287843v1_genomic (Scaffold) | SAMN05718081 | Human (urine) | USA | 2016 | NZ_MRBC01000076.1 | NONE |
| Enterobacter_hormaechei_GCF_003054595.1_ASM305459v1_genomic (Contig) | SAMN08160791 | Human (urine) | USA (New York: New York City) | 2013 | NZ_PNXR01000002.1 | IncHI2A_1,TrfA_1,IncHI2_1,*blaKPC-4_1,strA_1,aph(6)-Id_1,dfrA21_1,blaOXA-129_1,ant(3'')-Ia_1,sul1_5* |
| Enterobacter_hormaechei_GCF_003054635.1_ASM305463v1_genomic (Contig) | SAMN08160788 | Human (respiratory) | USA (New York: New York City) | 2014 | NZ_PNXT01000003.1 | IncHI2A_1,TrfA_1,IncHI2_1,*blaSHV-12_1,sul1_5,ere(A)_2,aac(3)-IIb_1,aac(6')-IIc_1,blaTEM-1A_4,aadA2_1,sul1_5,qnrB2_1,sul1_5,dfrA18_1,strA_1,aph(6)-Id_1,aph(3')-Ia_10* |
| Enterobacter_hormaechei_GCF_003227375.1_ASM322737v1_genomic (Contig) | SAMN08383101 | Animal (canine: urine) | Italy (Province of Padua: Padova) | 2016 | NZ_PVWV01000045.1 | NONE |
| Enterobacter_hormaechei_GCF_003260505.1_ASM326050v1_genomic (Contig) | SAMN07291541 | NA | NA | NA | NZ_QHCJ01000004.1 | IncHI2_1,TrfA_1,IncHI2A_1,*aadA2_1,sul1_5,qnrB2_1,sul1_5,dfrA18_1,strA_1,aph(6)-Id_1,catA1_1,dfrA8_1,ant(3'')-Ia_1,sul1_5,blaSHV-12_1,aph(3')-Ia_7,aadA2_1,sul1_5,qnrB2_1,sul1_5* |
| Enterobacter_hormaechei_GCF_003261215.1_ASM326121v1_genomic (Contig) | SAMN09425566 | Human | China (Shandong) | 2011 | NZ_QLNR01000066.1 | NONE |
| Enterobacter_hormaechei_GCF_003289325.1_ASM328932v1_genomic (Scaffold) | SAMN09435819 | Human (urine) | France | 2016 | NZ_QMCX01000153.1 | NONE |
| Enterobacter_hormaechei_GCF_003324425.1_ASM332442v1_genomic (Contig) | SAMN04014873 | NA | NA | NA | NZ_QMGH01000005.1 | IncHI2_1,TrfA_1,IncHI2A_1,*sul1_5,ere(A)_2,aac(3)-IIb_1,aac(6')-IIc_1,blaTEM-1B_1,catA2_1,qnrB2_1,sul1_5,dfrA18_1,strA_1,aph(6)-Id_1,aph(3')-Ia_10,sul2_2* |
| Enterobacter_hormaechei_GCF_003408595.1_ASM340859v1_genomic (Complete Genome) | SAMN04448499 | Human (rectal) | NA | 2015 | NZ_CP031575.1 (Plasmid pIncHI2-1502264) | IncHI2A_1,TrfA_1,IncHI2_1,*blaSHV-12_1,aph(6)-Id_1,strA_1,blaTEM-1B_1* |
| Enterobacter_hormaechei_GCF_003428425.1_ASM342842v1_genomic (Complete Genome) | SAMN08027258 | Human | China (Sichuan: Chengdu) | 2016 | NZ_CP031724.1 (Plasmid pCTXM9_020038) | IncHI2A_1,IncHI2_1,TrfA_1,*ant(2'')-Ia_1,aadA2_1,sul1_5,qnrA1_1,sul1_5,tet(A)_6,blaSHV-12_1,catA1_1,dfrA16_1,aadA2_1,sul1_5,blaCTX-M-9_1* |
| Enterobacter_hormaechei_GCF_003660125.1_ASM366012v1_genomic (Complete Genome) | SAMN10174734 | NA | Australia (New South Wales: Sydney) | 2007 | NZ_CP032842.1 (Plasmid pSPRC-Echo1) | IncHI2A_1,IncHI2_1,TrfA_1,*blaTEM-1B_1,blaSHV-12_1,sul2_2,sul1_5,ere(A)_2,aac(3)-IIb_1,aac(6')-IIc_1,sul1_5,qnrA1_1,sul1_5,dfrA18_1,strA_1,aph(6)-Id_1,tet(D)_1,catA2_1,blaSHV-12_1* |
| Enterobacter_hormaechei_GCF_003687945.1_ASM368794v1_genomic (Contig) | SAMN10252251 | NA | Poland | 2017 | NZ_RDWF01000125.1 | NONE |
| Enterobacter_hormaechei_GCF_003693465.1_ASM369346v1_genomic (Contig) | SAMN10252247 | NA | France | 2017 | NZ_RDRC01000150.1 | NONE |
| Enterobacter_hormaechei_GCF_003964445.1_ASM396444v1_genomic (Contig) | SAMN09845227 | Human | China (Sichuan: Chengdu) | 2018 | NZ_RXSF01000080.1 | NONE |
| Enterobacter_hormaechei_GCF_003965095.1_ASM396509v1_genomic (Contig) | SAMN08027254 | Human | China (Sichuan) | 2016 | NZ_RXPO01000068.1 | NONE |
| Enterobacter_hormaechei_GCF_003965465.1_ASM396546v1_genomic (Contig) | SAMN09845214 | Human | China (Sichuan: Chengdu) | 2016 | NZ_RXRS01000053.1 | *dfrA16_1* |
| Enterobacter_hormaechei_GCF_003965535.1_ASM396553v1_genomic (Contig) | SAMN08932722 | Human | China (Sichuan) | 2016 | NZ_RXQC01000072.1 | NONE |
| Enterobacter_hormaechei_GCF_003965635.1_ASM396563v1_genomic (Contig) | SAMN09845199 | Human | China (Sichuan: Chengdu) | 2018 | NZ_RXRE01000093.1 | NONE |
| Enterobacter_hormaechei_GCF_003965735.1_ASM396573v1_genomic (Contig) | SAMN08932721 | Human | China (Sichuan: Chengdu) | 2016 | NZ_RXQB01000098.1 | NONE |
| Enterobacter_hormaechei_GCF_003986785.1_ASM398678v1_genomic (Contig) | SAMN08932730 | Human | China (Sichuan: Chengdu) | 2014 | NZ_RXQK01000069.1 | NONE |
| Enterobacter_hormaechei_subsp._hoffmannii_ECNIH3_GCF_000750225.1_ASM75022v1_genomic (Complete Genome) | SAMN02713683 | NA | USA | 2011 | NZ_CP008899.1 (Plasmid pENT-8a4) | IncHI2A_1,IncHI2_1,TrfA_1,*sul2_2,blaSHV-12_1,catA2_1* |
| Enterobacter_hormaechei_subsp._hoffmannii_GCF_001472005.1_SMART_271.v1_genomic (Contig) | SAMN04252893 | Human | Taiwan | 2010 | NZ_LPQO01000038.1 | NONE |
| Enterobacter_hormaechei_subsp._hoffmannii_GCF_001526005.1_ASM152600v1_genomic (Contig) | SAMN04252894 | Human | Taiwan | 2010 | NZ_LREQ01000063.1 | NONE |
| Enterobacter_hormaechei_subsp._hoffmannii_GCF_002968455.1_ASM296845v1_genomic (Complete Genome) | SAMN07291508 | NA | NA | NA | NZ_CP027144.1 (Plasmid unnamed1) | IncHI2A_1,TrfA_1,IncHI2_1,*sul2_2,sul1_5,ere(A)_2,aac(3)-IIb_1,aac(6')-IIc_1,aadA2_1,sul1_5,dfrA18_1,strA_1,aph(6)-Id_1* |
| Enterobacter_hormaechei_subsp._hoffmannii_GCF_003688105.1_ASM368810v1_genomic (Contig) | SAMN10252253 | NA | Argentina | 2017 | NZ_RDWD01000195.1 | NONE |
| Enterobacter_hormaechei_subsp._hoffmannii_GCF_003688225.1_ASM368822v1_genomic (Contig) | SAMN10252235 | NA | Spain | 2017 | NZ_RDWT01000099.1 | *blaCTX-M-9_1* |
| Enterobacter_hormaechei_subsp._oharae_GCF_000803045.1_ASM80304v1_genomic (Contig) | SAMN03135815 | Human (rectal) | Brazil | 2012 | NZ_JSBO01000027.1 | IncHI2A_1 |
| Enterobacter_hormaechei_subsp._oharae_GCF_001054075.1_ASM105407v1_genomic (Scaffold) | SAMN03197164 | Human | USA (Washington) | NA | NZ_JVZA01000208.1 | NONE |
| Enterobacter_hormaechei_subsp._oharae_GCF_001471915.1_SMART_267.v1_genomic (Contig) | SAMN04252890 | Human | Spain | 2010 | NZ_LPQR01000052.1 | NONE |
| Enterobacter_hormaechei_subsp._oharae_GCF_001471975.1_SMART_268.v1_genomic (Contig) | SAMN04252891 | Human | Spain | 2010 | NZ_LPQQ01000061.1 | NONE |
| Enterobacter_hormaechei_subsp._oharae_GCF_001524915.1_ASM152491v1_genomic (Contig) | SAMN04430990 | Human | Australia | 2014 | NZ_LRJW01000114.1 | NONE |
| Enterobacter_hormaechei_subsp._steigerwaltii_GCF_000814125.3_ASM81412v3_genomic (Complete Genome) | SAMN03067414 | Human (bronchoalveolar lavage) | USA | 2009 | NZ_CP012170.1 (Plasmid p34977-263.138kb) | IncHI2_1,TrfA_1,IncHI2A_1,*aph(3')-Ia_10,aph(6)-Id_1,strA_1,dfrA18_1,sul1_5,qnrB2_1,sul1_5,aadA2_1,aac(6')-IIc_1,aac(3)-IIb_1,ere(A)_2,sul1_5,sul2_2,blaSHV-12_1* |
| Enterobacter_hormaechei_subsp._steigerwaltii_GCF_000957015.1_ASM95701v1_genomic (Contig) | SAMN03283674 | NA | USA (Florida) | 2012 | NZ_JZZD01000081.1 | NONE |
| Enterobacter_hormaechei_subsp._steigerwaltii_GCF_000957095.1_ASM95709v1_genomic (Contig) | SAMN03283647 | NA | USA (Florida) | 2012 | NZ_JZYZ01000044.1 | *blaCTX-M-9_1* |
| Enterobacter_hormaechei_subsp._steigerwaltii_GCF_000957165.1_ASM95716v1_genomic (Contig) | SAMN03283681 | NA | USA (Florida) | 2012 | NZ_JZYT01000057.1 | NONE |
| Enterobacter_hormaechei_subsp._steigerwaltii_GCF_000957445.1_ASM95744v1_genomic (Contig) | SAMN03283317 | NA | USA (New Jersey) | 2009 | NZ_LABB01000041.1 | NONE |
| Enterobacter_hormaechei_subsp._steigerwaltii_GCF_000957775.1_ASM95777v1_genomic (Contig) | SAMN03283639 | NA | USA (New York: New York City) | 2012 | NZ_JZZW01000052.1 | NONE |
| Enterobacter_hormaechei_subsp._steigerwaltii_GCF_000958025.1_ASM95802v1_genomic (Contig) | SAMN03283669 | NA | USA (Florida) | 2012 | NZ_JZZE01000065.1 | NONE |
| Enterobacter_hormaechei_subsp._steigerwaltii_GCF_000958095.1_ASM95809v1_genomic (Contig) | SAMN03283658 | NA | USA (Florida) | 2012 | NZ_JZYW01000019.1 | *ant(2'')-Ia_1,aadA2_1,sul1_5,blaCTX-M-9_1* |
| Enterobacter_hormaechei_subsp._steigerwaltii_GCF_001011725.1_ASM101172v1_genomic (Contig) | SAMN03495964 | Human | USA (North Carolina: Durham) | 2002 | NZ_LDCB01000074.1 | NONE |
| Enterobacter_hormaechei_subsp._steigerwaltii_GCF_001022425.1_ASM102242v1_genomic (Contig) | SAMN03732704 | Human | USA (North Carolina: Durham) | 2006 | NZ_LEDY01000029.1 | NONE |
| Enterobacter_hormaechei_subsp._steigerwaltii_GCF_001022515.1_ASM102251v1_genomic (Contig) | SAMN03732709 | Human | USA (North Carolina: Durham) | 2006 | NZ_LEDU01000036.1 | NONE |
| Enterobacter_hormaechei_subsp._steigerwaltii_GCF_001022605.1_ASM102260v1_genomic (Contig) | SAMN03732714 | Human | USA (North Carolina: Durham) | 2007 | NZ_LEDP01000038.1 | NONE |
| Enterobacter_hormaechei_subsp._steigerwaltii_GCF_001023375.1_ASM102337v1_genomic (Contig) | SAMN03732696 | Human | USA (North Carolina: Durham) | 2004 | NZ_LEEG01000035.1 | NONE |
| Enterobacter_hormaechei_subsp._steigerwaltii_GCF_001023395.1_ASM102339v1_genomic (Contig) | SAMN03732697 | Human | USA (North Carolina: Durham) | 2004 | NZ_LEEF01000028.1 | NONE |
| Enterobacter_hormaechei_subsp._steigerwaltii_GCF_001052955.1_ASM105295v1_genomic (Scaffold) | SAMN03197162 | Human | USA (Washington) | NA | NZ_JVZC01000077.1 | NONE |
| Enterobacter_hormaechei_subsp._steigerwaltii_GCF_001057845.1_ASM105784v1_genomic (Contig) | SAMN03197869 | Human | USA (Washington) | NA | NZ_JUXX01000066.1 | NONE |
| Enterobacter_hormaechei_subsp._steigerwaltii_GCF_001471255.1_SMART_1141.v1_genomic (Contig) | SAMN04252959 | Human | Hungary | 2013 | NZ_LPOB01000088.1 | NONE |
| Enterobacter_hormaechei_subsp._steigerwaltii_GCF_001472075.1_SMART_291.v1_genomic (Contig) | SAMN04252897 | Human | USA | 2010 | NZ_LPQL01000054.1 | NONE |
| Enterobacter_hormaechei_subsp._steigerwaltii_GCF_001472975.1_SMART_935.v1_genomic (Contig) | SAMN04252943 | Human | Puerto Rico | 2013 | NZ_LPOR01000030.1 | NONE |
| Enterobacter_hormaechei_subsp._steigerwaltii_GCF_001475405.1_SMART_1118.v1_genomic (Contig) | SAMN04252957 | Human | Romania | 2013 | NZ_LPOD01000035.1 | NONE |
| Enterobacter_hormaechei_subsp._steigerwaltii_GCF_001518415.1_ASM151841v1_genomic (Contig) | SAMN04407796 | Human | USA (North Carolina: Durham) | 2014 | NZ_LRCZ01000063.1 | NONE |
| Enterobacter_hormaechei_subsp._steigerwaltii_GCF_001524995.1_ASM152499v1_genomic (Contig) | SAMN04430989 | Human | Australia | 2014 | NZ_LRJX01000084.1 | NONE |
| Enterobacter_hormaechei_subsp._steigerwaltii_GCF_001526105.1_ASM152610v1_genomic (Contig) | SAMN04430999 | Human (urine) | Greece | 2014 | NZ_LRJN01000040.1 | NONE |
| Enterobacter_hormaechei_subsp._steigerwaltii_GCF_001526145.1_ASM152614v1_genomic (Contig) | SAMN04431000 | Human | Greece | 2014 | NZ_LRJM01000026.1 | NONE |
| Enterobacter_hormaechei_subsp._steigerwaltii_GCF_001631145.1_ASM163114v1_genomic (Contig) | SAMN04572597 | Human | USA (North Carolina: Durham) | 2012 | NZ_LVTY01000049.1 | NONE |
| Enterobacter_hormaechei_subsp._steigerwaltii_GCF_001631685.1_ASM163168v1_genomic (Contig) | SAMN04572593 | Human | USA (North Carolina: Durham) | 2012 | NZ_LVTU01000018.1 | NONE |
| Enterobacter_hormaechei_subsp._steigerwaltii_GCF_001631835.1_ASM163183v1_genomic (Contig) | SAMN04572690 | Human | USA (North Carolina: Durham) | 2013 | NZ_LVUO01000080.1 | NONE |
| Enterobacter_hormaechei_subsp._steigerwaltii_GCF_002184445.2_ASM218444v2_genomic (Contig) | SAMN06812668 | Human (blood) | USA | 2015 | NZ_NEYN02000039.1 | NONE |
| Enterobacter_hormaechei_subsp._steigerwaltii_GCF_002334865.1_ASM233486v1_genomic (Scaffold) | SAMD00089524 | Human | Japan (Kantō: Tokyo) | 2010 | NZ_BEGS01000081.1 | *blaCTX-M-9_1* |
| Enterobacter_hormaechei_subsp._steigerwaltii_GCF_002850625.1_ASM285062v1_genomic (Scaffold) | SAMN07816158 | Human | China (Sichuan: Chengdu) | 2017 | NZ_PDVT01000079.1 | NONE |
| Enterobacter_hormaechei_subsp._steigerwaltii_GCF_002936045.2_ASM293604v2_genomic (Contig) | SAMN08513492 | Human | USA (North Carolina) | 2014 | NZ_PTHV02000013.1 | NONE |
| Enterobacter_hormaechei_subsp._steigerwaltii_GCF_002936105.2_ASM293610v2_genomic (Contig) | SAMN08513484 | Human (urine) | USA (North Carolina) | 2014 | NZ_PTID02000021.1 | NONE |
| Enterobacter_hormaechei_subsp._xiangfangensis_GCF_000956725.1_ASM95672v1_genomic (Contig) | SAMN03283319 | NA | USA (New York: New York City) | 2011 | NZ_LAAS01000053.1 | NONE |
| Enterobacter_hormaechei_subsp._xiangfangensis_GCF_000956855.1_ASM95685v1_genomic (Contig) | SAMN03283629 | NA | USA (New York: New York City) | 2012 | NZ_LAAH01000058.1 | NONE |
| Enterobacter_hormaechei_subsp._xiangfangensis_GCF_000957085.1_ASM95708v1_genomic (Contig) | SAMN03283670 | NA | USA (Florida) | 2012 | NZ_JZZA01000053.1 | NONE |
| Enterobacter_hormaechei_subsp._xiangfangensis_GCF_000957135.1_ASM95713v1_genomic (Contig) | SAMN03283671 | NA | USA (Florida) | 2012 | NZ_JZYU01000054.1 | NONE |
| Enterobacter_hormaechei_subsp._xiangfangensis_GCF_000957595.1_ASM95759v1_genomic (Contig) | SAMN03283615 | NA | USA (New York: New York City) | 2012 | NZ_LAAL01000069.1 | NONE |
| Enterobacter_hormaechei_subsp._xiangfangensis_GCF_000958225.1_ASM95822v1_genomic (Contig) | SAMN03283682 | NA | USA (Michigan) | 2013 | NZ_JZYF01000076.1 | NONE |
| Enterobacter_hormaechei_subsp._xiangfangensis_GCF_001471215.1_SMART_1113.v1_genomic (Contig) | SAMN04252956 | Human | Croatia | 2013 | NZ_LPOE01000047.1 | NONE |
| Enterobacter_hormaechei_subsp._xiangfangensis_GCF_001472255.1_SMART_409.v1_genomic (Contig) | SAMN04252905 | Human | Spain | 2011 | NZ_LPQD01000050.1 | NONE |
| Enterobacter_hormaechei_subsp._xiangfangensis_GCF_001472455.1_SMART_562.v1_genomic (Contig) | SAMN04252916 | Human | Croatia | 2011 | NZ_LPPS01000083.1 | NONE |
| Enterobacter_hormaechei_subsp._xiangfangensis_GCF_001472685.1_SMART_723.v1_genomic (Contig) | SAMN04252928 | Human | Spain | 2012 | NZ_LPPG01000032.1 | NONE |
| Enterobacter_hormaechei_subsp._xiangfangensis_GCF_001472815.1_SMART_843.v1_genomic (Contig) | SAMN04252934 | Human | Croatia | 2012 | NZ_LPPA01000073.1 | NONE |
| Enterobacter_hormaechei_subsp._xiangfangensis_GCF_001473015.1_SMART_1001.v1_genomic (Contig) | SAMN04252944 | Human | Taiwan | 2013 | NZ_LPOQ01000095.1 | NONE |
| Enterobacter_hormaechei_subsp._xiangfangensis_GCF_001475415.1_SMART_1112.v1_genomic (Contig) | SAMN04252955 | Human | Croatia | 2013 | NZ_LPOF01000044.1 | NONE |
| Enterobacter_hormaechei_subsp._xiangfangensis_GCF_001518545.1_ASM151854v1_genomic (Contig) | SAMN04407779 | Human | USA (North Carolina: Durham) | 2010 | NZ_LRCK01000156.1 | NONE |
| Enterobacter_hormaechei_subsp._xiangfangensis_GCF_002177145.2_ASM217714v2_genomic (Contig) | SAMN06812647 | Human (blood) | USA (North Carolina) | 2015 | NZ_NFFB02000043.1 | NONE |
| Enterobacter_hormaechei_subsp._xiangfangensis_GCF_002936065.2_ASM293606v2_genomic (Contig) | SAMN08513488 | Human (respiratory) | USA (North Carolina) | 2014 | NZ_PTHZ02000074.1 | NONE |
| Enterobacter_hormaechei_subsp._xiangfangensis_GCF_002936085.2_ASM293608v2_genomic (Contig) | SAMN08513485 | Human (respiratory) | USA (North Carolina) | 2014 | NZ_PTIC02000036.1 | NONE |
| Enterobacter_hormaechei_subsp._xiangfangensis_GCF_002936125.2_ASM293612v2_genomic (Contig) | SAMN08513486 | Human (respiratory) | USA (North Carolina) | 2014 | NZ_PTIB02000077.1 | NONE |
| Enterobacter_hormaechei_subsp._xiangfangensis_GCF_002936775.2_ASM293677v2_genomic (Contig) | SAMN08513490 | Human (respiratory) | USA (North Carolina) | 2013 | NZ_PTHX02000002.1 | NONE |
| Enterobacter_hormaechei_subsp._xiangfangensis_GCF_002936795.2_ASM293679v2_genomic (Contig) | SAMN08513491 | Human (wound) | USA (North Carolina) | 2014 | NZ_PTHW02000062.1 | NONE |
| Enterobacter_hormaechei_subsp._xiangfangensis_GCF_002936835.2_ASM293683v2_genomic (Contig) | SAMN08513482 | Human (wound) | USA (Michigan) | 2015 | NZ_PTIF02000047.1 | NONE |
| Enterobacter_hormaechei_subsp._xiangfangensis_GCF_002936855.2_ASM293685v2_genomic (Contig) | SAMN08513487 | Human (respiratory) | USA (North Carolina) | 2014 | NZ_PTIA02000050.1 | NONE |
| Enterobacter_hormaechei_subsp._xiangfangensis_GCF_003382725.1_ASM338272v1_genomic (Complete Genome) | SAMN09071586 | Animal (canine) | USA (Ohio: Columbus) | 2016 | NZ_CP029248.1 (Plasmid pOSUEC_D) | IncHI2A_1,IncHI2_1,TrfA_1,*sul1_5,ant(3'')-Ia_1,blaOXA-129_1,dfrA21_1,aph(6)-Id_1,strA_1,tet(B)_2,blaKPC-4_1* |
| Enterobacter_hormaechei_subsp._xiangfangensis_GCF_003400705.1_ASM340070v1_genomic (Contig) | SAMN08623810 | Human (blood) | USA (California: Palo Alto) | 2015 | NZ_PXKP01000085.1 | NONE |
| Enterobacter_hormaechei_subsp._xiangfangensis_GCF_003586025.1_ASM358602v1_genomic (Complete Genome) | SAMN07988874 | Animal (canine: wound) | USA (Ohio: Columbus) | 2016 | NZ_CP024910.1 (Plasmid pOSUKPC4) | IncHI2A_1,IncHI2_1,TrfA_1,*strA_1,aph(6)-Id_1,dfrA21_1,blaOXA-129_1,ant(3'')-Ia_1,sul1_5,tet(B)_2,blaKPC-4_1* |
| Enterobacter_hormaechei_subsp._xiangfangensis_GCF_003964845.1_ASM396484v1_genomic (Contig) | SAMN10525013 | Human (sputum) | China (Sichuan: Chengdu) | 2018 | NZ_RXSS01000077.1 | NONE |
| Enterobacter_hormaechei_subsp._xiangfangensis_GCF_003977165.1_ASM397716v1_genomic (Contig) | SAMN10527291 | NA (blood) | Egypt | NA | NZ_RYXN01000098.1 | NONE |
| Enterobacter_kobei_GCF_001058415.1_ASM105841v1_genomic (Scaffold) | SAMN03197979 | Human | USA (Washington) | NA | NZ_JUTR01000067.1 | NONE |
| Enterobacter_kobei_GCF_001058605.1_ASM105860v1_genomic (Scaffold) | SAMN03198059 | Human | USA (Washington) | NA | NZ_JUQP01000043.1 | NONE |
| Enterobacter_kobei_GCF_001316705.2_ASM131670v2_genomic (Contig) | SAMN04011437 | Human (urine) | South Africa (KwaZulu-Natal: Durban) | 2013 | NZ_LJED02000199.1 | NONE |
| Enterobacter_kobei_GCF_001317225.2_ASM131722v2_genomic (Contig) | SAMN04011456 | Human (endotracheal aspirate) | South Africa (KwaZulu-Natal: Durban) | 2013 | NZ_LJEW02000010.1 | NONE |
| Enterobacter_kobei_GCF_001472595.1_SMART_635.v1_genomic (Contig) | SAMN04252923 | Human | Spain | 2012 | NZ_LPPL01000140.1 | NONE |
| Enterobacter_kobei_GCF_001729765.1_ASM172976v1_genomic (Complete Genome) | SAMN05581747 | Human (blood) | Japan (Nagano: Tatsuno) | NA | NZ_CP017181.1 (Chromosome) | IncFII(pECLA)_1_pECLA,blaACT-9_1,*fosA_1,oqxA_1,oqxB_1* |
| Enterobacter_kobei_GCF_002208285.1_ASM220828v1_genomic (Contig) | SAMN07234400 | Human (blood) | Australia (South Australia: Adelaide) | 2017 | NZ_NJCZ01000091.1 | NONE |
| Enterobacter_kobei_GCF_002208285.1_ASM220828v1_genomic (Contig) | SAMN07234400 | Human (blood) | Australia (South Australia: Adelaide) | 2017 | NZ_NJCZ01000093.1 | NONE |
| Enterobacter_kobei_GCF_002334635.1_ASM233463v1_genomic (Scaffold) | SAMD00089514 | Human | Japan (Kantō: Tokyo) | 2008 | NZ_BEGI01000089.1 | NONE |
| Enterobacter_kobei_GCF_002785705.1_ASM278570v1_genomic (Scaffold) | SAMN06767788 | Human (sputum) | China (Zhejiang: Hangzhou) | 2011 | NZ_NEEN01000051.1 | NONE |
| Enterobacter_kobei_GCF_002785725.1_ASM278572v1_genomic (Scaffold) | SAMN06767787 | Human (sputum) | China (Zhejiang: Hangzhou) | 2011 | NZ_NEEO01000050.1 | NONE |
| Enterobacter_kobei_GCF_002785745.1_ASM278574v1_genomic (Scaffold) | SAMN06767785 | Human (sputum) | China (Zhejiang: Hangzhou) | 2011 | NZ_NEEQ01000057.1 | NONE |
| Enterobacter_kobei_GCF_002785755.1_ASM278575v1_genomic (Scaffold) | SAMN06767784 | Human (sputum) | China (Zhejiang: Hangzhou) | 2011 | NZ_NEER01000063.1 | NONE |
| Enterobacter_kobei_GCF_002785785.1_ASM278578v1_genomic (Scaffold) | SAMN06767783 | Human | China (Zhejiang: Hangzhou) | NA | NZ_NEES01000010.1 | NONE |
| Enterobacter_kobei_GCF_002785795.1_ASM278579v1_genomic (Scaffold) | SAMN06767781 | Human (urine) | China (Zhejiang: Hangzhou) | 2010 | NZ_NEEU01000079.1 | NONE |
| Enterobacter_kobei_GCF_002785795.1_ASM278579v1_genomic (Scaffold) | SAMN06767781 | Human (urine) | China (Zhejiang: Hangzhou) | 2010 | NZ_NEEU01000070.1 | NONE |
| Enterobacter_kobei_GCF_002785905.1_ASM278590v1_genomic (Scaffold) | SAMN06767786 | Human (sputum) | China (Zhejiang: Hangzhou) | 2011 | NZ_NEEP01000070.1 | NONE |
| Enterobacter_kobei_GCF_002785945.1_ASM278594v1_genomic (Scaffold) | SAMN06767782 | Human | China (Zhejiang: Hangzhou) | NA | NZ_NEET01000054.1 | NONE |
| Enterobacter_kobei_GCF_002785945.1_ASM278594v1_genomic (Scaffold) | SAMN06767782 | Human | China (Zhejiang: Hangzhou) | NA | NZ_NEET01000027.1 | NONE |
| Enterobacter_kobei_GCF_003289725.1_ASM328972v1_genomic (Scaffold) | SAMN09435822 | Human (rectal) | France | 2017 | NZ_QMCU01000150.1 | NONE |
| Enterobacter_kobei_GCF_003665375.1_ASM366537v1_genomic (Complete Genome) | SAMN08027255 | Human | China (Sichuan: Chengdu) | 2017 | NZ_CP032893.1 (Plasmid p1_045523) | IncFIB(K)_1_Kpn3,IncFII(Yp)_1_Yersenia,IncFII(pRSB107)_1_pRSB107,*aadA2_1,sul1_5* |
| Enterobacter_mori_GCF_003986695.1_ASM398669v1_genomic (Contig) | SAMN08027256 | Human | China (Sichuan) | 2016 | NZ_RXPP01000065.1 | NONE |
| Enterobacter_roggenkampii_GCF_000957765.1_ASM95776v1_genomic (Contig) | SAMN03283637 | NA | USA (New York: New York City) | 2012 | NZ_JZZX01000051.1 | NONE |
| Enterobacter_roggenkampii_GCF_001022755.1_ASM102275v1_genomic (Contig) | SAMN03732722 | Human | USA (North Carolina: Durham) | 2008 | NZ_LEDI01000055.1 | NONE |
| Enterobacter_roggenkampii_GCF_001317055.2_ASM131705v2_genomic (Contig) | SAMN04011448 | Human (abdominal fluid) | South Africa (KwaZulu-Natal: Durban) | 2013 | NZ_LJEO02000051.1 | NONE |
| Enterobacter_roggenkampii_GCF_001472305.1_SMART_454.v1_genomic (Contig) | SAMN04252909 | Human | USA | 2011 | NZ_LPPZ01000071.1 | NONE |
| Enterobacter_roggenkampii_GCF_002162415.1_ASM216241v1_genomic (Contig) | SAMN06218064 | Human (blood) | USA | 2013 | NZ_NDIV01000004.1 | NONE |
| Enterobacter_sp._56-7_GCA_001898735.1_ASM189873v1_genomic (Scaffold)* | SAMN05660598 | Environmental (ammonium sulfate bioreactor) | South Africa (Western Cape: Cape Town) | 2014 | MKVD01000078.1 | NONE |
| Enterobacter_sp._BIDMC94_GCF_001037815.1_Ente_cloa_complex_BIDMC94_V1_genomic (Scaffold) | SAMN03280189 | Human | NA | 2014 | NZ_KQ089970.1 | NONE |
| Enterobacter_sp._BWH63_GCF_001037595.1_Ente_cloa_complex_BWH63_V1_genomic (Scaffold) | SAMN03280179 | Human | USA | 2014 | NZ_KQ089880.1 | IncHI2_1,TrfA_1,IncHI2A_1,*blaKPC-4_1,sul1_5,ant(3'')-Ia_1,blaOXA-129_1,dfrA21_1,aph(6)-Id_1,strA_1,blaKPC-4_1* |
| Enterobacter_sp._BWH_37_GCF_000534495.1_Ente_cloa_complex_BWH_37_V1_genomic (Scaffold) | SAMN02138600 | NA (rectal) | NA | NA | NZ_KI973198.1 | IncHI2A_1,TrfA_1,IncHI2_1,*tet(B)_2,strA_1,aph(6)-Id_1,dfrA21_1,blaOXA-129_1,ant(3'')-Ia_1,sul1_5,blaKPC-4_1* |
| Enterobacter_sp._DKU_NT_01_GCA_002142555.1_ASM214255v1_genomic (Chromosome) | SAMN06844203 | Food (soybean) | South Korea (South Chungcheong Province) | 2017 | CP021137.1 (Chromosome) | *fosA_7,blaACT-7_1,oqxA_1,oqxB_1* |
| Enterobacter_sp._E20_GCF_000801755.2_ASM80175v2_genomic (Complete Genome) | SAMN03099801 | Environmental (glyphosate polluted soil) | China (Zhejiang) | 2010 | NZ_CP012999.1 (Chromosome) | *blaACT-10_1,fosA_1,oqxA_1,oqxB_1* |
| Enterobacter_sp._K66-74_GCF_001463035.1_ASM146303v1_genomic (Contig) | SAMN04158285 | Human (rectal) | Norway | 2010 | NZ_LNHC01000057.1 | NONE |
| Enterobacter_sp._MGH_15_GCF_000534555.1_Ente_cloa_complex_MGH_15_V1_genomic (Scaffold) | SAMN02138555 | NA (respiratory) | NA | NA | NZ_KI973218.1 | IncHI2_1,TrfA_1,IncHI2A_1,*aadA2_1,catA2_1,blaTEM-1B_1,sul2_2,blaSHV-12_1,aph(3')-Ia_10,aph(6)-Id_1,strA_1,dfrA18_1,sul1_5,qnrB2_1,sul1_5,qnrB2_1* |
| Enterobacteriaceae_bacterium_w17_GCF_003336325.1_ASM333632v1_genomic (Complete Genome) | SAMN09667311 | Human (abdominal) | China (Zhejiang: Hangzhou) | NA | NZ_CP031102.1 (Plasmid pW17-1) | IncHI2_1,TrfA_1,IncHI2A_1 |
| Escherichia_coli_BIDMC_15_GCF_000522245.1_Esch_coli_BIDMC_15_V2_genomic (Scaffold) | SAMN02138638 | NA (abdominal) | NA | NA | NZ_KI929740.1 | *sul2_2,blaKPC-2_1* |
| Escherichia_coli_BIDMC_17A_GCF_000522225.1_Esch_coli_BIDMC_17A_V2_genomic (Scaffold) | SAMN02138640 | NA (blood) | NA | NA | NZ_KI929722.1 | *sul2_2* |
| Escherichia_coli_BIDMC_17B_GCF_000522205.1_Esch_coli_BIDMC_17B_V2_genomic (Scaffold) | SAMN02138641 | NA (peritoneal fluid) | NA | NA | NZ_KI929708.1 | IncHI2A_1,TrfA_1,IncHI2_1,*aac(6')-IIc_1,aac(3)-IIb_1,ere(A)_2,sul1_5,blaSHV-12_1,sul2_2* |
| Escherichia_coli_BIDMC_19A_GCF_000522185.1_Esch_coli_BIDMC_19A_V2_genomic (Scaffold) | SAMN02138646 | NA (bronchoalveolar lavage) | NA | NA | NZ_KI929699.1 | IncHI2A_1,TrfA_1,IncHI2_1,*blaKPC-2_1,aac(6')-IIc_1,aac(3)-IIb_1,ere(A)_2,sul1_5,blaSHV-12_1,sul2_2* |
| Escherichia_coli_BIDMC_19B_GCF_000522165.1_Esch_coli_BIDMC_19B_V2_genomic (Scaffold) | SAMN02138647 | NA (urine) | NA | NA | NZ_KI929692.1 | IncHI2A_1,TrfA_1,IncHI2_1,*aac(6')-IIc_1,aac(3)-IIb_1,ere(A)_2,sul1_5,blaSHV-12_1,sul2_2* |
| Escherichia_coli_BIDMC_19C_GCF_000474825.1_Esch_coli_BIDMC_19C_V1_PacBio_genomic (Contig) | SAMN02138648 | NA (urine) | NA | NA | NZ_AXLI01000004.1 | IncHI2A_1,TrfA_1,IncHI2_1,*blaKPC-2_1,aac(6')-IIc_1,aac(3)-IIb_1,ere(A)_2,sul1_5,blaSHV-12_1,sul2_2* |
| Escherichia_coli_BIDMC_2B_GCF_000522305.1_Esch_coli_BIDMC_2B_V2_genomic (Scaffold) | SAMN02138622 | NA (blood) | NA | NA | NZ_KI929776.1 | IncHI2A_1,TrfA_1,IncHI2_1,*aac(6')-IIc_1,aac(3)-IIb_1,ere(A)_2,sul1_5,blaSHV-12_1,sul2_2* |
| Escherichia_coli_BIDMC_3_GCF_000522285.1_Esch_coli_BIDMC_3_V2_genomic (Scaffold) | SAMN02138623 | NA (abdominal incision) | NA | NA | NZ_KI929769.1 | IncHI2A_1,TrfA_1,IncHI2_1,*aac(6')-IIc_1,aac(3)-IIb_1,ere(A)_2,sul1_5,blaSHV-12_1,sul2_2* |
| Escherichia_coli_BIDMC_43a_GCF_000522105.1_Esch_coli_BIDMC_43a_V1_genomic (Scaffold) | SAMN02356581 | Human (blood) | USA (Massachusetts: Boston) | NA | NZ_KI929670.1 | IncHI2A_1,TrfA_1,IncHI2_1,*blaKPC-2_1,aac(6')-IIc_1,aac(3)-IIb_1,ere(A)_2,sul1_5,blaSHV-12_1,sul2_2* |
| Escherichia_coli_BIDMC_43b_GCF_000522085.1_Esch_coli_BIDMC_43b_V1_genomic (Scaffold) | SAMN02356582 | Human (blood) | USA (Massachusetts: Boston) | NA | NZ_KI929662.1 | IncHI2_1,TrfA_1,IncHI2A_1,*blaSHV-12_1,sul1_5,ere(A)_2,aac(3)-IIb_1,aac(6')-IIc_1,blaKPC-2_1,sul2_2* |
| Escherichia_coli_BIDMC_6_GCF_000522405.1_Esch_coli_BIDMC_6_V2_genomic (Scaffold) | SAMN02138626 | NA (bile) | NA | NA | NZ_KI929809.1 | IncHI2A_1,TrfA_1,IncHI2_1,*aac(6')-IIc_1,aac(3)-IIb_1,ere(A)_2,sul1_5,blaSHV-12_1,sul2_2* |
| Escherichia_coli_BIDMC_82_GCF_000633675.1_Esch_coli_BIDMC_82_V1_genomic (Scaffold) | SAMN02581401 | Human (urine) | NA | 2013 | NZ_KK214247.1 | NONE |
| Escherichia_coli_BIDMC_9_GCF_000522265.1_Esch_coli_BIDMC_9_V2_genomic (Scaffold) | SAMN02138630 | NA (urine) | NA | NA | NZ_KI929755.1 | IncHI2_1,TrfA_1,IncHI2A_1,*sul2_2,blaSHV-12_1,sul1_5,ere(A)_2,aac(3)-IIb_1,aac(6')-IIc_1* |
| Escherichia_coli_GCA_003891015.1_PDT000343901.1_genomic (Contig)* | SAMN09633793 | Environmental | United Kingdom | 2017 | RTSZ01000082.1 | NONE |
| Escherichia_coli_GCA_900607665.1_ecoli009_genomic (Contig)* | SAMEA4916050 | Human | Switzerland (Basel-Stadt: Basel) | 2010 | UWVU01000002.1 | IncHI2_1,TrfA_1,IncHI2A_1,*sul1_5,ant(3'')-Ia_1,blaVIM-1_1,catA1_1,blaACC-1_2,aph(6)-Id_1,strA_1,catA2_1,aadA2_1,sul1_5,qnrB2_1,sul1_5,dfrA18_1,strA_1,aph(6)-Id_1,sul1_9,aph(3')-Ia_10* |
| Escherichia_coli_GCA_900607975.1_ecoli015_genomic (Contig)* | SAMEA4916082 | Human | Switzerland (Basel-Stadt: Basel) | 2011 | UWWX01000002.1 | IncHI2A_1,TrfA_1,IncHI2_1,*aph(3')-Ia_10,aph(3')-Ia_10,sul1_9,aph(6)-Id_1,strA_1,dfrA18_1,sul1_5,qnrB2_1,sul1_5,aadA2_1,catA2_1,strA_1,aph(6)-Id_1,blaACC-1_2,catA1_1,blaVIM-1_1,ant(3'')-Ia_1,sul1_5,aph(3')-Ia_10,aph(3')-Ia_10* |
| Escherichia_coli_GCA_900608175.1_ecoli025_genomic (Contig)* | SAMEA4916102 | Human | Switzerland (Basel-Stadt: Basel) | 2010 | UWXQ01000002.1 | IncHI2A_1,TrfA_1,IncHI2_1,*aph(3')-Ia_10,aph(3')-Ia_10,sul1_9,aph(6)-Id_1,strA_1,dfrA18_1,sul1_5,qnrB2_1,sul1_5,aadA2_1,catA2_1,strA_1,aph(6)-Id_1,blaACC-1_2,catA1_1,blaVIM-1_1,ant(3'')-Ia_1,sul1_5,aph(3')-Ia_10,aph(3')-Ia_10* |
| Escherichia_coli_GCF_001182885.1_ASM118288v1_genomic (Scaffold) | SAMN03491847 | Human (sputum) | Australia (Queensland: Brisbane) | 2014 | NZ_LFXU01000076.1 | NONE |
| Escherichia_coli_GCF_002152995.1_ASM215299v1_genomic (Scaffold) | SAMN04521847 | Human | USA (Massachusetts: Boston) | 2015 | NZ_NGRV01000004.1 | TrfA_1,IncHI2_1 |
| Escherichia_coli_GCF_002516635.1_ASM251663v1_genomic (Contig) | SAMN04279485 | Animal (equine: bladder) | USA (Colorado) | 2007 | NZ_NOGH01000062.1 | NONE |
| Escherichia_coli_GCF_002537655.1_ASM253765v1_genomic (Contig) | SAMN05439490 | Environmental | USA (Minnesota) | 2011 | NZ_NMDK01000130.1 | NONE |
| Escherichia_coli_GCF_002542525.1_ASM254252v1_genomic (Contig) | SAMN05439445 | Environmental | USA (Minnesota) | 2010 | NZ_NMEQ01000120.1 | NONE |
| Escherichia_coli_GCF_002545085.1_ASM254508v1_genomic (Contig) | SAMN04902855 | Animal (wild boar: large intestine) | USA (South Dakota) | 2012 | NZ_NNYP01000106.1 | NONE |
| Escherichia_coli_GCF_003338885.1_ASM333888v1_genomic (Contig) | SAMN09579993 | Animal (wild turkey) | Australia | 2009 | NZ_QOGU01000094.1 | NONE |
| Escherichia_coli_GCF_004004795.1_ASM400479v1_genomic (Contig) | SAMN10644704 | Environmental (siphon) | France (Nouvelle-Aquitaine: Limoges) | 2017 | NZ_RZVV01000232.1 | NONE |
| Escherichia_coli_GCF_900007315.1_VAP_LM33_genomic (Scaffold) | SAMEA3496963 | NA | France | NA | NZ_LN874955.1 | IncHI2_1,TrfA_1,IncHI2A_1,*blaTEM-1C_5,dfrA18_1,strA_1,aph(6)-Id_1,catA2_1,blaSHV-12_1,aac(6')-IIc_1,aac(3)-IIb_1,ere(A)_2,sul1_5* |
| Escherichia_coli_GCF_900500325.1_68A_genomic (Contig)^h^ | SAMEA4811195 | Human (rectal) | France (Île-de-France: Paris) | 2018 | NZ_UILU01000010.1 | IncHI2A_1,TrfA_1,IncHI2_1 |
| Escherichia_coli_KTE156_GCF_000351965.1_Esch_coli_KTE156_V1_genomic (Scaffold) | SAMN00854682 | Human | Denmark | 2010 | NZ_KB733420.1 | IncHI2_1,TrfA_1,IncHI2A_1 |
| Escherichia_coli_UMEA_3318-1_GCF_000461235.1_Esch_coli_UMEA_3318-1_V1_genomic (Scaffold) | SAMN01885952 | Human (urine) | Sweden | 1995 | NZ_KE702348.1 | IncHI2A_1,TrfA_1,IncHI2_1 |
| Franconibacter_helveticus_513_GCF_000485945.1_Ehel513.sqn_genomic (Contig) | SAMN02364109 | Food (fruit powder) | Switzerland | 2007 | NZ_AXDK01000097.1 | NONE |
| Klebsiella_michiganensis_GCF_001038305.1_ASM103830v1_genomic (Scaffold) | SAMN03737905 | Human (urine) | USA (California: San Francisco) | 2009 | NZ_LDZM01000054.1 | NONE |
| Klebsiella_michiganensis_GCF_002186735.1_ASM218673v1_genomic (Contig) | SAMN05960896 | Human (blood) | Nigeria | 2012 | NZ_NFVT01000122.1 | NONE |
| Klebsiella_oxytoca_GCF_001022115.1_ASM102211v1_genomic (Complete Genome) | SAMN03733750 | Human (urine) | USA (Virginia: Charlottesville) | 2010 | NZ_CP011617.1 (Plasmid pCAV1335-115) | IncFIB(K)_1_Kpn3,*sul1_5,aadA2_1,aph(3')-Ia_10,aph(6)-Id_1,strA_1,dfrA18_1,sul1_5,qnrB2_1* |
| Klebsiella_oxytoca_GCF_001022295.1_ASM102229v1_genomic (Complete Genome) | SAMN03733663 | Human (perirectal) | USA (Virginia: Charlottesville) | 2009 | NZ_CP011596.1 (Plasmid pCAV1099-114) | IncFIB(K)_1_Kpn3,*sul1_5,aadA2_1,aph(3')-Ia_10,aph(6)-Id_1,strA_1,dfrA18_1,sul1_5,qnrB2_1* |
| Klebsiella_oxytoca_GCF_001870185.1_ASM187018v1_genomic (Complete Genome) | SAMN03733631 | Human (blood) | USA (Virginia: Charlottesville) | 2007 | NZ_CP017930.1 (Plasmid pCAV1015-114) | IncFIB(K)_1_Kpn3,*sul1_5,qnrB2_1,sul1_5,dfrA18_1,strA_1,aph(6)-Id_1,aph(3')-Ia_10,aadA2_1* |
| Klebsiella_oxytoca_GCF_003416995.1_ASM341699v1_genomic (Contig) | SAMN08963300 | Environmental (river) | Sweden (Närke: Örebro) | 2015 | NZ_QDDT01000058.1 | NONE |
| Klebsiella_oxytoca_GCF_003417005.1_ASM341700v1_genomic (Contig) | SAMN08963299 | Human (urine) | Sweden (Närke: Örebro) | 2014 | NZ_QDDU01000060.1 | NONE |
| Klebsiella_oxytoca_GCF_003417035.1_ASM341703v1_genomic (Contig) | SAMN08963297 | Human (tissue) | Sweden (Närke: Örebro) | 2014 | NZ_QDDW01000056.1 | NONE |
| Klebsiella_pneumoniae_GCA_001901745.1_ASM190174v1_genomic (Contig)* | SAMN05178492 | Human (blood) | South Korea (Seoul Capital Area: Seoul) | 2010 | LYPV01000097.1 | NONE |
| Klebsiella_pneumoniae_GCF_001482645.1_ASM148264v1_genomic (Scaffold) | SAMN04124436 | Human (pleural fluid) | Denmark | 2014 | NZ_LLJP01000056.1 | NONE |
| Klebsiella_pneumoniae_GCF_002288845.1_ASM228884v1_genomic (Scaffold) | SAMN06204663 | Human (rectal) | Singapore | NA | NZ_NSMB01000045.1 | NONE |
| Klebsiella_pneumoniae_GCF_002752865.1_ASM275286v1_genomic (Scaffold) | SAMN07807166 | Human (rectal) | Singapore | 2014 | NZ_PEHD01000045.1 | NONE |
| Klebsiella_pneumoniae_GCF_003175675.1_ASM317567v1_genomic (Contig) | SAMD00115685 | Human | NA | 2000 | NZ_BGLF01000094.1 | NONE |
| Klebsiella_pneumoniae_GCF_003316315.1_ASM331631v1_genomic (Contig) | SAMN08374133 | Human (feces) | Australia (Western Australia: Perth) | 2015 | NZ_PPSS01000068.1 | NONE |
| Klebsiella_pneumoniae_GCF_003402125.1_ASM340212v1_genomic (Contig) | SAMN08963296 | Human (feces) | Sweden (Närke: Örebro) | 2014 | NZ_QDDX01000084.1 | NONE |
| Klebsiella_pneumoniae_GCF_900608205.1_kpneu037_genomic (Contig) | SAMEA4916105 | Human | Switzerland (Basel-Stadt: Basel) | 2015 | NZ_UWXT01000002.1 | IncHI2_1,TrfA_1,IncHI2A_1,IncHI2_1,TrfA_1,*blaTEM-1B_1,dfrA18_1,strA_1,aph(6)-Id_1,catA2_1,aph(3')-Ia_1,mph(A)_2,mph(A)_1,sul1_5,ARR-3_4,catB3_1,blaOXA-1_1,aac(6')Ib-cr_1,blaSHV-12_1,sul1_5,blaDHA-7_1,qnrB4_1,sul1_5,ere(A)_2,aac(3)-IIb_1,aac(6')-IIc_1,blaTEM-1B_1,aac(6')Ib-cr_1,dfrA18_1,strA_1,aph(6)-Id_1,catA2_1,aph(3')-Ia_1,mph(A)_2,mph(A)_1,sul1_5,ARR-3_4,catB3_1,blaOXA-1_1,blaSHV-12_1,sul1_5,ant(3'')-Ia_1,blaOXA-1_1,blaSHV-12_1,sul1_5,blaDHA-7_1,qnrB4_1,sul1_5,ere(A)_2,aac(3)-IIb_1,aac(6')-IIc_1* |
| Klebsiella_pneumoniae_subsp._pneumoniae_GCF_002936565.2_ASM293656v2_genomic (Contig) | SAMN08513429 | Human (urine) | USA (Ohio) | 2013 | NZ_PTGO02000119.1 | NONE |
| Klebsiella_quasipneumoniae_GCF_003583875.1_ASM358387v1_genomic (Scaffold) | SAMN10025409 | Human (rectal) | Brazil (Minas Gerais: Uberlândia) | 2015 | NZ_QXXP01000129.1 | NONE |
| Klebsiella_quasipneumoniae_subsp._similipneumoniae_GCF_900080215.1_KQS06HV_PRJEB13450_wgs.embl_genomic (Contig) | SAMEA3940169 | Human (urine) | Mexico | 2006 | NZ_FKLR01000041.1 | NONE |
| Leclercia_sp._LSNIH1_GCF_002902985.1_ASM290298v1_genomic (Complete Genome) | SAMN06040403 | Environmental (wastewater and/or sludge) | USA (Maryland: Bethesda) | 2016 | NZ_CP026168.1 (Plasmid pLEC-b38d) | NONE |
| Leclercia_sp._LSNIH2_GCF_002920235.1_ASM292023v1_genomic (Contig) | SAMN06040407 | Environmental (wastewater and/or sludge) | USA (Maryland: Bethesda) | 2016 | NZ_PQKC01000046.1 | NONE |
| Leclercia_sp._LSNIH5_GCF_002919545.1_ASM291954v1_genomic (Contig) | SAMN06040410 | Environmental (wastewater and/or sludge) | USA (Maryland: Bethesda) | 2016 | NZ_PQKB01000041.1 | NONE |
| Phytobacter_ursingii_GCF_001022135.1_ASM102213v1_genomic (Complete Genome) | SAMN03733692 | Human (perirectal) | USA (Virginia: Charlottesville) | 2009 | NZ_CP011601.1 (Plasmid pCAV1151-296) | IncHI2A_1,TrfA_1,IncHI2_1,*catA2_1,aac(6')-IIc_1,aac(3)-IIb_1,ere(A)_2,sul1_5,sul2_2,blaTEM-1B_1,catA2_1,aadA2_1,sul1_5,dfrA18_1,strA_1,aph(6)-Id_1,aph(3')-Ia_10* |
| Pluralibacter_gergoviae_GCF_002151975.1_ASM215197v1_genomic (Scaffold) | SAMN04521932 | Human | USA (Massachusetts: Boston) | 2015 | NZ_NGRU01000003.1 | IncHI2A_1,TrfA_1,IncHI2_1,*catA2_1,blaSHV-12_1,sul2_2,aadA2_1,sul1_5,dfrA18_1,strA_1,aph(6)-Id_1,tet(D)_1,blaTEM-1A_4* |
| Pluralibacter_gergoviae_GCF_002152975.1_ASM215297v1_genomic (Scaffold) | SAMN04521922 | Human | USA (Massachusetts: Boston) | 2015 | NZ_NGRT01000004.1 | *tet(D)_1* |
| Proteus_mirabilis_WGLW6_GCF_000297815.1_Prot_mira_WGLW6_V1_genomic (Scaffold) | SAMN02463961 | Human | NA | NA | NZ_JH815505.1 | *cat_1,aph(6)-Id_1,strA_1,dfrA18_1,sul1_5,aadA2_1,catA2_1,sul2_2* |
| Raoultella_sp._YJ_GCF_002108615.1_ASM210861v1_genomic (Contig) | SAMN06209158 | Environmental (contaminated soil) | China (Zhejiang: Wenzhou) | 2016 | NZ_MTEG01000076.1 | NONE |
| Salmonella_enterica_GCA_003747185.1_PDT000398670.1_genomic (Contig)* | SAMN10339238 | NA | USA | NA | RLTW01000072.1 | NONE |
| Salmonella_enterica_GCA_003747185.1_PDT000398670.1_genomic (Contig)* | SAMN10339238 | NA | USA | NA | RLTW01000096.1 | NONE |
| Salmonella_enterica_GCA_003767365.1_PDT000399580.1_genomic (Contig)* | SAMN10346702 | Animal (swine) | USA (Minnesota) | 2018 | RMYC01000125.1 | NONE |
| Salmonella_enterica_GCA_003767365.1_PDT000399580.1_genomic (Contig)* | SAMN10346702 | Animal (swine) | USA (Minnesota) | 2018 | RMYC01000099.1 | NONE |
| Salmonella_enterica_GCA_003785745.1_PDT000400412.1_genomic (Contig)* | SAMN10359268 | NA | USA | NA | ROVE01000055.1 | NONE |
| Salmonella_enterica_GCA_003785745.1_PDT000400412.1_genomic (Contig)* | SAMN10359268 | NA | USA | NA | ROVE01000082.1 | NONE |
| Salmonella_enterica_GCA_003874275.1_PDT000292474.2_genomic (Contig)* | SAMN08452516 | Environmental (pet food) | USA (Washington) | 2016 | RSQS01000061.1 | NONE |
| Salmonella_enterica_GCA_003895815.1_PDT000344458.1_genomic (Contig)* | SAMN09631611 | Food (sausage) | USA (Utah) | 2017 | RUFV01000118.1 | NONE |
| Salmonella_enterica_GCA_003896135.1_PDT000382611.1_genomic (Contig)* | SAMN10102234 | NA | USA | NA | RUCL01000124.1 | NONE |
| Salmonella_enterica_GCA_003896135.1_PDT000382611.1_genomic (Contig)* | SAMN10102234 | NA | USA | NA | RUCL01000155.1 | NONE |
| Salmonella_enterica_GCF_001473915.1_Salmonella_enterica_CVM_43839_v1.0_genomic (Contig) | SAMN02912008 | Human | USA | 2012 | NZ_JYTE01000069.1 | NONE |
| Salmonella_enterica_GCF_002036945.1_ASM203694v1_genomic (Contig) | SAMN03357124 | NA | NA | NA | NZ_MXCG01000048.1 | NONE |
| Salmonella_enterica_GCF_003325055.1_ASM332505v1_genomic (Complete Genome) | SAMN06045172 | NA | NA | NA | NZ_CP030186.1 (Plasmid pSA20094620.1) | TrfA_1,IncHI2_1,IncHI2A_1,*blaCTX-M-15_23,tet(D)_1,dfrA18_1,sul1_5,ere(A)_2,aac(3)-IIb_1,aac(6')-IIc_1,catA2_1,sul2_2,sul2_11,catA2_1* |
| Salmonella_enterica_GCF_003429915.1_ASM342991v1_genomic (Contig) | SAMN08057887 | Environmental | USA (North Carolina) | 2016 | NZ_QUUD01000033.1 | NONE |
| Salmonella_enterica_GCF_003429945.1_ASM342994v1_genomic (Contig) | SAMN08057883 | Environmental | USA (North Carolina) | 2016 | NZ_QUUE01000019.1 | NONE |
| Salmonella_enterica_GCF_003429975.1_ASM342997v1_genomic (Contig) | SAMN08057880 | Environmental (animal feed) | USA (North Carolina) | 2016 | NZ_QUTZ01000028.1 | NONE |
| Salmonella_enterica_GCF_003430205.1_ASM343020v1_genomic (Contig) | SAMN08057876 | Environmental | USA (North Carolina) | 2016 | NZ_QUUA01000027.1 | NONE |
| Salmonella_enterica_GCF_003430225.1_ASM343022v1_genomic (Contig) | SAMN08057888 | Environmental | USA (North Carolina) | 2016 | NZ_QUUC01000027.1 | NONE |
| Salmonella_enterica_GCF_003430385.1_ASM343038v1_genomic (Contig) | SAMN09695778 | Environmental | USA (Iowa) | 2016 | NZ_QUZX01000019.1 | NONE |
| Salmonella_enterica_GCF_900241525.1_STY150_genomic (Contig) | SAMEA104429256 | Animal (swine) | Italy | 2012 | NZ_OEUJ01000104.1 | NONE |
| Salmonella_enterica_GCF_900242465.1_STY5_genomic (Contig) | SAMEA104429347 | Human | Italy | 2013 | NZ_OEXX01000070.1 | NONE |
| Salmonella_enterica_GCF_900242515.1_STY6_genomic (Contig) | SAMEA104429354 | Human | Italy | 2013 | NZ_OEYK01000077.1 | NONE |
| Salmonella_enterica_subsp._enterica_GCA_003869185.1_PDT000031463.3_genomic (Contig)* | SAMN02403374 | NA (turkey) | USA (Virginia) | 2006 | RSGR01000007.1 | IncHI2A_1,TrfA_1,IncHI2_1 |
| Salmonella_enterica_subsp._enterica_GCA_003870555.1_PDT000094695.3_genomic (Contig)* | SAMN02777831 | Food (ground turkey) | USA (Connecticut) | 2004 | RSHP01000061.1 | NONE |
| Salmonella_enterica_subsp._enterica_GCA_003870555.1_PDT000094695.3_genomic (Contig)* | SAMN02777831 | Food (ground turkey) | USA (Connecticut) | 2004 | RSHP01000106.1 | NONE |
| Salmonella_enterica_subsp._enterica_GCF_002999175.1_ASM299917v1_genomic (Complete Genome) | SAMN08475067 | Food (minced meat) | Germany | 2015 | NZ_CP026661.1 (Plasmid pSE15-SA01028) | IncHI2A_1,IncHI2_1,TrfA_1,*blaACC-1_2,sul1_9,aph(6)-Id_1,strA_1,sul1_5,ant(3'')-Ia_1,ere(A)_1,sul1_5,ant(3'')-Ia_1,blaVIM-1_1,catA1_1* |
| Salmonella_enterica_subsp._enterica_serovar_4_12_i_-_GCF_003077195.1_ASM307719v1_genomic (Contig) | SAMN08514455 | Animal (equine: feces) | USA (Texas: College Station) | 2015 | NZ_QDLK01000212.1 | NONE |
| Salmonella_enterica_subsp._enterica_serovar_4_5_12_i_-_GCF_003067295.1_ASM306729v1_genomic (Contig) | SAMN08897994 | Animal (swine) | USA (Texas: College Station) | 2015 | NZ_QBHG01000062.1 | NONE |
| Salmonella_enterica_subsp._enterica_serovar_4_5_12_i_-_GCF_003067325.1_ASM306732v1_genomic (Contig) | SAMN08897995 | Animal (swine) | USA (Texas: College Station) | 2015 | NZ_QBHH01000102.1 | NONE |
| Salmonella_enterica_subsp._enterica_serovar_4_5_12_i_-_GCF_003067345.1_ASM306734v1_genomic (Contig) | SAMN08897996 | Animal (swine) | USA (Texas: College Station) | 2015 | NZ_QBHI01000065.1 | NONE |
| Salmonella_enterica_subsp._enterica_serovar_Agona_GCF_003117295.1_ASM311729v1_genomic (Contig) | SAMN08514249 | Animal (equine: feces) | USA (Texas: College Station) | 2007 | NZ_QDTF01000046.1 | NONE |
| Salmonella_enterica_subsp._enterica_serovar_Agona_GCF_003118725.1_ASM311872v1_genomic (Contig) | SAMN08514381 | Animal (equine: feces) | USA (Texas: College Station) | 2011 | NZ_QDOG01000048.1 | NONE |
| Salmonella_enterica_subsp._enterica_serovar_Agona_GCF_003120155.1_ASM312015v1_genomic (Contig) | SAMN08514310 | Animal (equine: feces) | USA (Texas: College Station) | 2008 | NZ_QDQW01000046.1 | NONE |
| Salmonella_enterica_subsp._enterica_serovar_Agona_GCF_003120635.1_ASM312063v1_genomic (Contig) | SAMN08514288 | Animal (equine: feces) | USA (Texas: College Station) | 2008 | NZ_QDRS01000050.1 | NONE |
| Salmonella_enterica_subsp._enterica_serovar_Anatum_GCF_003120995.1_ASM312099v1_genomic (Contig) | SAMN08514267 | Animal (equine: feces) | USA (Texas: College Station) | 2008 | NZ_QDSN01000089.1 | NONE |
| Salmonella_enterica_subsp._enterica_serovar_Concord_GCF_002062415.1_ASM206241v1_genomic (Contig) | SAMN02368733 | NA | NA | NA | NZ_MXWU01000054.1 | *blaCTX-M-9_1* |
| Salmonella_enterica_subsp._enterica_serovar_Concord_GCF_002065885.1_ASM206588v1_genomic (Contig) | SAMN02368547 | Human | Ethiopia | 2011 | NZ_MYDK01000046.1 | NONE |
| Salmonella_enterica_subsp._enterica_serovar_Concord_GCF_003030265.1_ASM303026v1_genomic (Complete Genome) | SAMN08663446 | NA | Czech Republic | NA | NZ_CP028197.1 (Plasmid pGMI14-002_1) | IncHI2A_1,TrfA_1,IncHI2_1,IncA/C2_1,*catA2_1,blaTEM-1B_1,blaSHV-12_1,aac(6')-IIc_1,aac(3)-IIb_1,ere(A)_2,sul1_5,sul2_2,aph(6)-Id_1,strA_1,dfrA18_1,sul1_5,qnrB2_1,tet(D)_1,catA2_1,floR_2,tet(A)_6,aph(6)-Id_1,aph(3'')-Ib_5,sul2_2,aac(6')-IIc_1,aac(3)-IIb_1,ere(A)_2,sul1_5,aph(6)-Id_1,strA_1,dfrA18_1* |
| Salmonella_enterica_subsp._enterica_serovar_Corvallis_GCF_003071545.1_ASM307154v1_genomic (Complete Genome) | SAMN08685598 | Animal (wild bird) | Germany | 2012 | NZ_CP027678.1 (Plasmid pSE12-01738-1) | IncHI2_1,TrfA_1,IncHI2A_1 |
| Salmonella_enterica_subsp._enterica_serovar_Cubana_GCF_002057115.1_ASM205711v1_genomic (Contig) | SAMN02368951 | Human | USA (New York) | 2010 | NZ_MYTG01000195.1 | NONE |
| Salmonella_enterica_subsp._enterica_serovar_Cubana_str._CFSAN002050_GCF_000430125.1_ASM43012v1_genomic (Complete Genome) | SAMN01832088 | Food (alfalfa sprouts) | USA (Arizona) | 2012 | NC_021819.1 (Plasmid unnamed2) | NONE |
| Salmonella_enterica_subsp._enterica_serovar_Heidelberg_GCF_001272375.1_Salmonella_enterica_CVM_N51271_v1.0_genomic (Contig) | SAMN03894362 | Food (ground turkey) | USA (Maryland) | 2013 | NZ_LIOC01000042.1 | NONE |
| Salmonella_enterica_subsp._enterica_serovar_Heidelberg_GCF_001479265.1_Salmonella_enterica_CVM_N31844-SQ_v1.0_genomic (Contig) | SAMN02699244 | Food (ground turkey) | USA (Maryland) | 2011 | NZ_JYVR01000050.1 | *aac(3)-VIa_1,ant(3'')-Ia_1* |
| Salmonella_enterica_subsp._enterica_serovar_Heidelberg_GCF_001690135.1_ASM169013v1_genomic (Complete Genome) | SAMN05263546 | Animal (equine) | Canada (Ontario) | 2009 | NZ_CP016526.1 (Plasmid p09-036813-1A_261) | IncHI2_1,TrfA_1,IncHI2A_1,*blaTEM-1B_1,catA2_1,aph(6)-Id_1,strA_1,dfrA18_1,sul1_5,qnrB2_1,sul1_5,ere(A)_2,aac(3)-IIb_1,aac(6')-IIc_1,aph(3')-Ia_10* |
| Salmonella_enterica_subsp._enterica_serovar_Infantis_GCF_002110505.1_ASM211050v1_genomic (Contig) | SAMN05912852 | Food (minced pork meat) | Germany | 2015 | NZ_MLFV01000046.1 | NONE |
| Salmonella_enterica_subsp._enterica_serovar_Infantis_GCF_002110515.1_ASM211051v1_genomic (Contig) | SAMN05912850 | Animal (swine) | Germany | 2016 | NZ_MLFW01000062.1 | NONE |
| Salmonella_enterica_subsp._enterica_serovar_Mbandaka_GCF_001481155.1_Salmonella_enterica_CVM_N42487-R_v1.0_genomic (Contig) | SAMN02699356 | Food (chicken breast) | USA (New Mexico) | 2012 | NZ_JZAA01000048.1 | NONE |
| Salmonella_enterica_subsp._enterica_serovar_Montevideo_GCF_001276695.1_ASM127669v1_genomic (Contig) | SAMN03339653 | NA | NA | NA | NZ_JZTM01000036.1 | NONE |
| Salmonella_enterica_subsp._enterica_serovar_Montevideo_GCF_001276905.1_ASM127690v1_genomic (Contig) | SAMN03339660 | NA | NA | NA | NZ_JZTT01000015.1 | NONE |
| Salmonella_enterica_subsp._enterica_serovar_Newport_GCF_001278315.1_ASM127831v1_genomic (Complete Genome) | SAMN03744303 | Animal (equine: feces) | USA (Pennsylvania: Kennett Square) | 2003 | NZ_CP012599.1 (Chromosome) | IncHI2_1,TrfA_1,IncHI2A_1,IncA/C2_1,*blaCMY-2_1,blaCMY-2_1,sul2_2,aph(3'')-Ib_5,aph(6)-Id_1,tet(A)_6,floR_2,blaTEM-1B_1,catA2_1,tet(D)_1,aadA2_1,sul1_5,qnrB2_1,sul1_5,dfrA18_1,strA_1,aph(6)-Id_1,sul1_5,ere(A)_2,aac(3)-IIb_1,aac(6')-IIc_1,aph(3')-Ia_10,aac(6')-Iaa_1,aph(3')-Ia_10,aph(3')-Ia_10,aph(3')-Ia_10,blaSHV-12_1,sul2_2,blaCMY-2_1* |
| Salmonella_enterica_subsp._enterica_serovar_Newport_GCF_002058605.1_ASM205860v1_genomic (Contig) | SAMN02368948 | Human | USA (Pennsylvania) | 2010 | NZ_MYTK01000397.1 | NONE |
| Salmonella_enterica_subsp._enterica_serovar_Rubislaw_GCF_003077475.1_ASM307747v1_genomic (Contig) | SAMN08514441 | Animal (equine: feces) | USA (Texas: College Station) | 2014 | NZ_QDLY01000040.1 | NONE |
| Salmonella_enterica_subsp._enterica_serovar_Rubislaw_GCF_003119455.1_ASM311945v1_genomic (Contig) | SAMN08514345 | Animal (equine: feces) | USA (Texas: College Station) | 2010 | NZ_QDPN01000047.1 | NONE |
| Salmonella_enterica_subsp._enterica_serovar_Rubislaw_GCF_003119635.1_ASM311963v1_genomic (Contig) | SAMN08514336 | Animal (equine: feces) | USA (Texas: College Station) | 2009 | NZ_QDPW01000060.1 | NONE |
| Salmonella_enterica_subsp._enterica_serovar_Saintpaul_GCF_001240225.1_Salmonella_enterica_CVM_N43456_v1.0_genomic (Contig) | SAMN03894065 | Food (ground turkey) | USA (Colorado) | 2013 | NZ_LHDT01000059.1 | NONE |
| Salmonella_enterica_subsp._enterica_serovar_Saintpaul_GCF_001240335.1_Salmonella_enterica_CVM_N43461_v1.0_genomic (Contig) | SAMN03894070 | Food (ground turkey) | USA (Louisiana) | 2013 | NZ_LHDY01000061.1 | NONE |
| Salmonella_enterica_subsp._enterica_serovar_Saintpaul_GCF_001241665.1_Salmonella_enterica_CVM_N45394_v1.0_genomic (Contig) | SAMN03894139 | Food (ground turkey) | USA (Louisiana) | 2013 | NZ_LHFY01000043.1 | NONE |
| Salmonella_enterica_subsp._enterica_serovar_Saintpaul_GCF_001242115.1_Salmonella_enterica_CVM_N45926_v1.0_genomic (Contig) | SAMN03894160 | Food (ground turkey) | USA (Connecticut) | 2013 | NZ_LHGQ01000039.1 | NONE |
| Salmonella_enterica_subsp._enterica_serovar_Saintpaul_GCF_001242655.1_Salmonella_enterica_CVM_N45953_v1.0_genomic (Contig) | SAMN03894184 | Food (ground turkey) | USA (New Mexico) | 2013 | NZ_LHHN01000058.1 | NONE |
| Salmonella_enterica_subsp._enterica_serovar_Saintpaul_GCF_001243085.1_Salmonella_enterica_CVM_N46823_v1.0_genomic (Contig) | SAMN03894202 | Food (ground turkey) | USA (Colorado) | 2013 | NZ_LHIC01000060.1 | NONE |
| Salmonella_enterica_subsp._enterica_serovar_Saintpaul_GCF_001243195.1_Salmonella_enterica_CVM_N46830_v1.0_genomic (Contig) | SAMN03894209 | Food (ground turkey) | USA (Louisiana) | 2013 | NZ_LHIH01000050.1 | NONE |
| Salmonella_enterica_subsp._enterica_serovar_Saintpaul_GCF_001243205.1_Salmonella_enterica_CVM_N46832_v1.0_genomic (Contig) | SAMN03894211 | Food (ground turkey) | USA (Louisiana) | 2013 | NZ_LHII01000048.1 | NONE |
| Salmonella_enterica_subsp._enterica_serovar_Saintpaul_GCF_001243905.1_Salmonella_enterica_CVM_N47719_v1.0_genomic (Contig) | SAMN03894249 | Food (ground turkey) | USA (New Mexico) | 2013 | NZ_LHJN01000065.1 | NONE |
| Salmonella_enterica_subsp._enterica_serovar_Saintpaul_GCF_001245335.1_Salmonella_enterica_CVM_N50423_v1.0_genomic (Contig) | SAMN03894303 | Food (ground turkey) | USA (Minnesota) | 2013 | NZ_LHLI01000050.1 | NONE |
| Salmonella_enterica_subsp._enterica_serovar_Saintpaul_GCF_001247105.1_Salmonella_enterica_CVM_N51291_v1.0_genomic (Contig) | SAMN03894382 | Food (ground turkey) | USA (New Mexico) | 2013 | NZ_LHOA01000057.1 | NONE |
| Salmonella_enterica_subsp._enterica_serovar_Saintpaul_GCF_001477905.1_Salmonella_enterica_CVM_N29360-SQ_v1.0_genomic (Contig) | SAMN02699202 | Food (ground turkey) | USA (Colorado) | 2011 | NZ_JYUB01000054.1 | NONE |
| Salmonella_enterica_subsp._enterica_serovar_Saintpaul_GCF_001478155.1_Salmonella_enterica_CVM_N31414-SQ_v1.0_genomic (Contig) | SAMN02699240 | Food (ground turkey) | USA (Tennessee) | 2011 | NZ_JYVN01000080.1 | NONE |
| Salmonella_enterica_subsp._enterica_serovar_Saintpaul_GCF_001478225.1_Salmonella_enterica_CVM_N32750-SQ_v1.0_genomic (Contig) | SAMN02699255 | Food (ground turkey) | USA (Georgia) | 2011 | NZ_JYWC01000060.1 | NONE |
| Salmonella_enterica_subsp._enterica_serovar_Saintpaul_GCF_001478245.1_Salmonella_enterica_CVM_N32763-SQ_v1.0_genomic (Contig) | SAMN02699258 | Food (ground turkey) | USA (New Mexico) | 2011 | NZ_JYWF01000049.1 | NONE |
| Salmonella_enterica_subsp._enterica_serovar_Saintpaul_GCF_001478745.1_Salmonella_enterica_CVM_N29369-R_v1.0_genomic (Contig) | SAMN02699206 | Food (ground turkey) | USA (New Mexico) | 2011 | NZ_JYUF01000046.1 | NONE |
| Salmonella_enterica_subsp._enterica_serovar_Saintpaul_GCF_001478765.1_Salmonella_enterica_CVM_N29377-SQ_v1.0_genomic (Contig) | SAMN02699207 | Food (ground turkey) | USA (New Mexico) | 2011 | NZ_JYUG01000055.1 | NONE |
| Salmonella_enterica_subsp._enterica_serovar_Saintpaul_GCF_001479205.1_Salmonella_enterica_CVM_N31415-SQ_v1.0_genomic (Contig) | SAMN02699241 | Food (ground turkey) | USA (Tennessee) | 2011 | NZ_JYVO01000104.1 | NONE |
| Salmonella_enterica_subsp._enterica_serovar_Saintpaul_GCF_001479275.1_Salmonella_enterica_CVM_N31846-SQ_v1.0_genomic (Contig) | SAMN02699245 | Food (pork chop) | USA (Maryland) | 2011 | NZ_JYVS01000023.1 | NONE |
| Salmonella_enterica_subsp._enterica_serovar_Saintpaul_GCF_002030545.1_ASM203054v1_genomic (Contig) | SAMN02368003 | Food (ground turkey) | USA (Tennessee) | 2011 | NZ_MXFE01000059.1 | NONE |
| Salmonella_enterica_subsp._enterica_serovar_Saintpaul_GCF_002031105.1_ASM203110v1_genomic (Contig) | SAMN02367985 | Food (ground turkey) | USA (New York) | 2011 | NZ_MXFT01000051.1 | NONE |
| Salmonella_enterica_subsp._enterica_serovar_Saintpaul_GCF_002032665.1_ASM203266v1_genomic (Contig) | SAMN02367984 | Food (ground turkey) | USA (New York) | 2011 | NZ_MXFU01000075.1 | NONE |
| Salmonella_enterica_subsp._enterica_serovar_Schwarzengrund_GCF_001478525.1_Salmonella_enterica_CVM_N41900-SQ_v1.0_genomic (Contig) | SAMN02699330 | Food (ground turkey) | USA (Connecticut) | 2012 | NZ_JYZA01000079.1 | NONE |
| Salmonella_enterica_subsp._enterica_serovar_Schwarzengrund_GCF_003600105.1_ASM360010v1_genomic (Scaffold) | SAMN10083767 | Human (feces) | USA (Kansas) | 2006 | NZ_QZEM01000116.1 | NONE |
| Salmonella_enterica_subsp._enterica_serovar_Senftenberg_GCF_001276745.1_ASM127674v1_genomic (Contig) | SAMN03339654 | NA | NA | NA | NZ_JZTN01000033.1 | NONE |
| Salmonella_enterica_subsp._enterica_serovar_Senftenberg_GCF_001276765.1_ASM127676v1_genomic (Contig) | SAMN03339655 | NA | NA | NA | NZ_JZTO01000021.1 | NONE |
| Salmonella_enterica_subsp._enterica_serovar_Senftenberg_GCF_001276775.1_ASM127677v1_genomic (Contig) | SAMN03339656 | NA | NA | NA | NZ_JZTP01000011.1 | NONE |
| Salmonella_enterica_subsp._enterica_serovar_Senftenberg_GCF_001276925.1_ASM127692v1_genomic (Contig) | SAMN03339662 | NA | NA | NA | NZ_JZTV01000007.1 | NONE |
| Salmonella_enterica_subsp._enterica_serovar_Senftenberg_str._361154004_GCF_000483995.1_ASM48399v1_genomic (Contig) | SAMN02415177 | NA | NA | NA | NZ_AYDO01000050.1 | NONE |
| Salmonella_enterica_subsp._enterica_serovar_Senftenberg_str._361154004_GCF_003073495.1_ASM307349v1_genomic (Complete Genome) | SAMN01816110 | Food (shelled pistachios) | USA (California) | 2009 | NZ_CP029037.1 (Plasmid unnamed) | IncHI2A_1,TrfA_1,IncHI2_1 |
| Salmonella_enterica_subsp._enterica_serovar_Typhimurium_GCF_002091095.1_ASM209109v1_genomic (Scaffold) | SAMN04394777 | Human (feces) | USA (Washington) | 2010 | NZ_NAAN01000063.1 | NONE |
| Serratia_marcescens_GCA_002738185.1_ASM273818v1_genomic (Scaffold) | SAMN07452559 | Human (urine) | Romania (Cluj: Cluj-Napoca) | 2015 | PEHB01000007.1 | *aph(6)-Id_1,strA_1,dfrA18_1,sul1_5,ere(A)_2,aac(3)-IIb_1,aac(6')-IIc_1,blaSHV-12_1,blaTEM-1B_1* |
| Serratia_marcescens_GCF_002250685.1_ASM225068v1_genomic (Scaffold) | SAMN07414881 | Human (lavage) | Austria (Carinthia: Klagenfurt) | 2017 | NZ_NPIX01000080.1 | NONE |
| Serratia_marcescens_GCF_900108835.1_SM1978_genomic (Contig) | SAMEA4524859 | NA | NA | NA | NZ_FNXW01000270.1 | NONE |
| Shigella_sp._GCA_003517755.1_ASM351775v1_genomic (Scaffold)* | SAMN08019495 | NA | NA | NA | DOZY01000196.1 | NONE |
| Superficieibacter_electus_GCF_002915515.1_ASM291551v1_genomic (Contig) | SAMN07405752 | Environmental (hospital ICU nurse call button) | Pakistan | 2016 | NZ_PQGE01000068.1 | NONE |
| Superficieibacter_electus_GCF_002915575.1_ASM291557v1_genomic (Contig) | SAMN07405751 | Environmental (hospital ICU bedside rail) | Pakistan | 2016 | NZ_PQGD01000067.1 | NONE |

^a^Assembly level corresponds to assembly level reported by NCBI (i.e., one of Complete Genome, Chromosome, Scaffold, or Contig); assemblies marked with an asterisk (*) had been excluded from RefSeq

^b^Isolation source derived solely from metadata available in the corresponding BioSample; each assembly was assigned to one of the following categories using BioSample metadata: Animal, Environmental, Food, Human, or NA (not available), with additional specific isolation source in parentheses

^c^Country of isolation derived solely from metadata available in the corresponding BioSample, with additional specific information (e.g., region, city) in parentheses; assemblies with no geographic information reported were designated by NA (not available)

^d^Year of isolation derived solely from metadata available in the corresponding BioSample; assemblies with no collection/isolation date reported were designated by NA (not available)

^e^NCBI Accession number of contig on which *mcr*-9 was detected using translated nucleotide blast (tblastn; C. Camacho, et al., BMC Bioinformatics 10:421, 2009, doi: 10.1186/1471-2105-10-421) as implemented in BTyper version 2.3.2 (L.M. Carroll, J. Kovac, R.A. Miller, and M. Wiedmann, Appl Environ Microbiol 83(17): e01096-17, 2017, doi: 10.1128/AEM.01096-17); for contigs that were designated as either a Plasmid or Chromosome in NCBI’s Assembly database (e.g., for assemblies belonging to the Complete Genome Assembly level), the name of the corresponding plasmid or chromosome is in parentheses

^f^Plasmid replicons and antimicrobial resistance (AMR) genes detected on the contig on which *mcr*-9 was detected. ABRicate version 0.8 (https://github.com/tseemann/abricate) was used with minimum identity thresholds of 80 and 75% and minimum coverage thresholds of 60 and 50%, with the PlasmidFinder (A. Carattoli, et al., Antimicrob Agents Chemother 58(7): 3895-3903, 2014, doi: 10.1128/AAC.02412-14) and ResFinder (E. Zankari, et al., J Antimicrob Chemother 67(11): 2640-2644, 2012, doi: 10.1093/jac/dks261) databases, respectively. If no plasmid replicons or AMR genes were detected, a designation of “NONE” is given

^g^*Enterobacter asburiae* assembly GCF_000965825.1 was reported to belong to a set of colistin resistant Enterobacter genomes (https://www.ncbi.nlm.nih.gov/bioproject/271006)

^h^*Escherichia coli* assembly GCF_900500325.1 was reported to be that of colistin resistant strain 68A; *mcr*-1 to -8 were reported to be absent from the genome, and no missense/nonsense mutations in *pmrAB* were observed (A.S. Bourrel, et al., J Antimicrob Chemother dkz090, 2019, doi: 10.1093/jac/dkz090)
